# Inhibiting Liver‐Derived C3 Protein Rescues Anesthesia/Surgery‐Induced Cognitive Impairment, Synaptic Disorders, and Microglial Phagocytosis

**DOI:** 10.1002/advs.202502034

**Published:** 2025-11-12

**Authors:** Qianqian Wu, Peilin Cong, Zhouxiang Li, Yuxin Zhang, Huanghui Wu, Qian Zhang, Yawei Li, Li Tian, Qingyuan Miao, Yinggang Zheng, Hui Zhang, Qian Chen, Enduo Feng, Xinyang Li, Zheping Chen, Dong‐Xin Wang, Xinwei Huang, Lize Xiong

**Affiliations:** ^1^ Shanghai Key Laboratory of Anesthesiology and Brain Functional Modulation Clinical Research Center for Anesthesiology and Perioperative Medicine Translational Research Institute of Brain and Brain‐Like Intelligence Department of Anesthesiology and Perioperative Medicine Shanghai Fourth People's Hospital School of Medicine Tongji University Shanghai 200434 China; ^2^ Department of Anesthesiology Peking University First Hospital Beijing 100034 China

**Keywords:** blood protein, cognitive impairment, complement C3, postoperative cognitive dysfunction, surgery

## Abstract

Identifying peripheral proteins having therapeutic effects on cognitive impairment could provide beneficial insights into the prevention and treatment of cognition‐related disorders, including postoperative cognitive dysfunction (POCD) that is a common postoperative cognitive impairment mainly caused by anesthesia/surgery. Here, proteomic and transcriptomic analyses in multiple organs from humans and POCD mice are conducted to identify potential peripheral targets for anesthesia/surgery‐induced cognitive impairment. The results show that anesthesia/surgery can disrupt the blood‐brain barrier (BBB) and promote the release of hepatogenous C3 protein into the blood. This surgical dual factors ultimately drove C3 to cross the damaged BBB and selectively colocalize with C3aR in the hippocampus. Anesthesia/surgery‐induced C3 upregulation in the liver is associated with hypomethylation of C3 promoter. Inhibiting hepatogenous C3 is demonstrated to salvage the anesthesia/surgery‐induced cognitive impairment, structural and functional injury of synapse, and C3aR‐mediated microglial phagocytosis. Perioperative alterations in serum C3 protein in surgical patients are related to POCD, showing potential for predicting this disorder. This study emphasizes that peripheral C3 is a promising target for the prevention and therapy, and a potential biomarker for predicting cognitive impairment, and confirms that the liver mediates anesthesia/surgery‐induced cognitive impairment.

## Graphical abstract

This study provides evidence that anesthesia/surgery can induce the BBB breakdown and promote the release of hepatogenous C3 protein into the blood. This surgical dual factors ultimately drove C3 to cross the damaged BBB and selectively colocalize with C3aR in the hippocampus, which results in structural and functional injury of synapse, C3aR‐mediated microglial phagocytosis, and cognitive impairment.

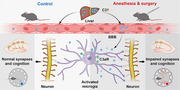

## 1 Introduction

Cognitive impairment is commonly observed in elderly individuals and patients with various neurological disorders with impairment of learning, memory, and sensory functions.^[^ ^1^, ^2^ ^]^ Anesthesia/surgery‐induced cognitive impairment (i.e., postoperative cognitive dysfunction (POCD) or perioperative neurocognitive disorder (PND)) is one common postoperative complication that occurs in ≈10–54% of surgical patients within first few weeks after surgery, increasing the incidence of poor quality of life, functional disability, mortality, and long‐term Alzheimer's disease (AD).^[^ ^3^, ^4^ ^]^ The anesthesia/surgery‐induced cognitive impairment will bring more serious challenges to society and families with the rapid growth of the surgical population.^[^ ^5^, ^6^ ^]^ To identify potential therapeutic targets involved in the pathogenesis of this disorder and determining biomarkers that have the potential to predict postoperative cognitive impairment in the surgical population is beneficial to the treatment or prevention of this disorder. At present, numerous studies have been mainly carried out in animal models and clinical samples, mainly focusing on the central nervous system (CNS), however, the underlying pathogenesis of this disorder is still inconclusive. In recent years, the increasing epidemiological evidence suggests that elderly surgical patients with chronic diseases in peripheral organs (such as hepatitis) may be more susceptible to POCD than other surgical population,^[^ ^7^, ^8^ ^]^ which provide us with a reasonable guess as to whether peripheral organs mediate the susceptibility to cognitive impairment.

Blood proteins are actively secreted or released into the blood by organs, having direct or indirect regulatory effects on the brain, and the liver is the prominent source of blood proteins in the human body.^[^ ^9^ ^]^ Mounting evidence reveals that blood proteins or metabolic molecules control communications from peripheral organs to the brain.^[^ ^9^, ^10^, ^11^, ^12^, ^13^, ^14^, ^15^, ^16^, ^17^ ^]^ Their concentrations are affected by lifestyle interventions and aging, and their imbalance in the blood can lead to cognitive impairment and the onset of neurological diseases.^[^ ^11^, ^14^, ^16^, ^17^ ^]^ Given the impacts of blood proteins on the CNS, they could be regarded as promising therapeutic targets to avoid cognitive impairment via enhancing cognitive resilience. To fulfill this potential, it is critical to discover those important blood proteins and their source organs affected by harmful factors of cognitive dysfunction such as anesthesia/surgery, as only a limited number of such proteins have been identified to be associated with cognitive impairment.

Complement component (C3), an essential immune regulatory factor, plays a crucial role in immune responses, particularly in the elimination of pathogens and the regulation of inflammation.^[^ ^18^ ^]^ Previous studies have indicated that endogenous C3 protein was excessively activated in the brains of patients with AD and in relevant models, and contributed to the progression of AD pathology and cognitive impairment.^[^ ^19^, ^20^, ^21^ ^]^ Nevertheless, it remains unclear whether peripheral C3 protein could influence the CNS and cognitive function.

In this study, we first performed proteomic or transcriptomic analyses in multiple organs from human and POCD model mice and determined the impacts of anesthesia/surgery on liver‐derived blood proteins, including C3. Subsequently, protein and transcription assessments at different postoperative time points in multiple organs of POCD mice showed that anesthesia/surgery resulted in the increases of C3 protein in the liver, serum, and hippocampus, without influence on *C3* transcription levels in the hippocampus and other peripheral organs. Bulk‐RNA and targeted bisulfite sequencing were harnessed to determine that anesthesia/surgery‐induced C3 upregulation in the liver was associated with hypomethylation of CpG sites in *C3* gene promoter. Moreover, transmission electron microscope (TEM) and dynamic tracking of C3 revealed that peripheral C3 can cross the CNS to bind to C3aR under the premise of BBB injury. Furthermore, we demonstrated that inhibition of hepatogenous C3 rescued anesthesia/surgery‐induced cognitive impairment, structural and functional injury of synapse, and C3aR‐mediated microglial phagocytosis. Finally, perioperative changes in serum C3 in surgical patients were found to be linked to postoperative cognitive impairment. Our study highlights peripheral C3 as a promising target for the prevention and therapy, as well as a biomarker for predicting cognitive dysfunction, and confirms that the liver mediates anesthesia/surgery‐induced cognitive impairment.

## 2 Results

### 2.1 Anesthesia/Surgery Alters the Proteomic Landscape of the Liver and Plasma in Mice

**Figure 1:**
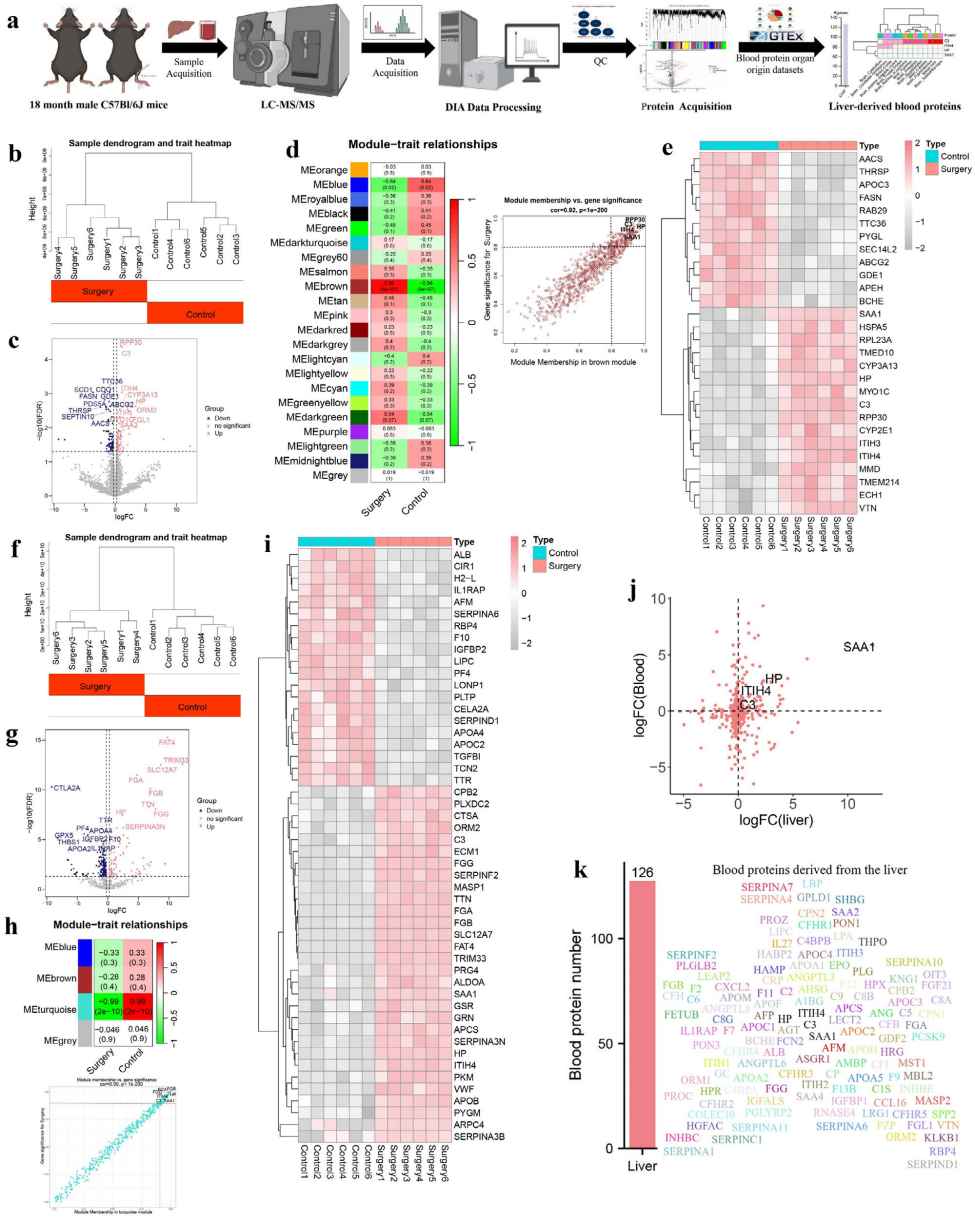
Identification of liver‐derived blood proteins disturbed by anesthesia/surgery in aged mice. a) Flow diagram of proteome study for the liver and plasma between control and surgery mice (n = 6 per group). b) Sample dendrogram and trait heatmap (unsupervised clustering) exhibiting distinct separation in liver proteins by two groups. c) Volcano plot illustrating differential proteins in the liver between control and surgery mice. d) Identification of the module (brown) most relevant to anesthesia/surgery in the liver using Weighted Gene Co‐expression Network Analysis (WGCNA). e) Heatmap showing the surgery‐disturbed overlapped proteins identified by both differential analysis and WGCNA in the liver. f) Sample dendrogram and trait heatmap exhibiting distinct separation in plasma proteins by two groups. g) Volcano plot illustrating differential proteins in the plasma between control and surgery mice. h) Identification of the module (turquoise) most relevant to anesthesia/surgery in the plasma utilizing WGCNA. i) Heatmap illustrating the surgery‐disturbed overlapped proteins identified by both differential analysis and WGCNA in the plasma. j) Scatter plot illustrating the surgery‐disturbed overlapped proteins in both the liver and plasma. k) Human blood proteins C3, HP, ITIH4, and SAA1 are mainly derived from the human liver which is the dominant sources of 126 blood proteins. This assessment was performed based on the Human Protein Atlas (https://www.proteinatlas.org/). Laparotomy was used as a surgical method under anesthesia in mice. Differential protein analysis was conducted by limma. Proteins with |log (fold change) | > 0.25 and Benjamini‐Hochberg adjusted *P* value < 0.05 were regarded as differential proteins.

To begin to characterize the proteomic changes in the liver and plasma caused by surgical trauma, we performed protein measurement in paired plasma and liver from control mice and surgery mice (aged 18 months, n = 6 per group) (**Figure** **1**a). For the liver, unsupervised clustering exhibited distinct separation by two groups in proteins (Figure 1b). Differential analysis identified 157 differential proteins between two groups (Figure 1c). Weighted Gene Co‐expression Network Analysis (WGCNA) determined the brown module mostly relevant to surgery (Cor = 0.92, *P* < 1E‐200) (Figure 1d), containing 12 downregulated and 16 upregulated differential proteins that had the strongest association with surgery group (|Cor| > 0.90, 3.89E‐09 < *P* < 5.10E‐06) and this module (|Cor| > 0.90, 4.66E‐08 < *P* < 4.77E‐05) (Figure 1e; Table S1, Supporting Information). The same analyses were further conducted in the plasma. We found that two groups also showed distinct separation in proteins (Figure 1f). A total of 212 proteins between two groups were identified by differential analysis (Figure 1g). WGCNA revealed that the turquoise module was mostly associated with surgery group (Cor = 0.99, *P* < 1E‐200) (Figure 1h), including 20 downregulated and 30 upregulated differential proteins having the strongest relationship with surgery group (|Cor| > 0.90, 5.79E‐06 < *P* < 5.79E‐05) and this module (|Cor| > 0.90, 7.03E‐06 < *P* < 6.35E‐05) (Figure 1i; Table S2, Supporting Information). Finally, four proteins (C3, HP, ITIH4, and SAA1) were identified to be upregulated in the both liver and plasma of surgery mice compared to control mice, showing a consistent positive association with surgery group (Figure 1j). The sample‐pairs correlation analysis for the four proteins revealed that their levels between the liver and plasma had a strong positive correlation (0.74 < R < 0.85, *P* < 0.008) (Figure S1a–d, Supporting Information), suggesting a possibility that those proteins in the plasma come at least in part from the liver.

To determine the organ source of the above proteins in the blood, we assessed their enrich and transcriptome atlas of their corresponding coding genes in human organs based on GTEx and HPA databases. A human secretome by Uhlén *et.al*.^[^ ^9^ ^]^ identified 780 proteins that were predicted to translocate to peripheral blood, having systemic impacts on the human body, as opposed to proteins that are leaky products of cell death and end up in the bloodstream due to disease causes. Those secretome proteins were defined as blood proteins. Of which, the coding genes of 247 proteins showed a tissue‐restricted expression in 26 human organs.^[^ ^9^ ^]^ Through assessing organ distribution of these proteins in this platform (HPA database, https://www.proteinatlas.org/), we determined that the liver was the organ with the highest amount of blood proteins, secreting at least 126 proteins into the blood, including C3, HP, ITIH4, and SAA1(Figure 1k; Table S3, Supporting Information). In addition, we downloaded the primary RNA Seq datasets from GTEx database (https://www.gtexportal.org/), including 13 brain regions and 29 peripheral organs of 11 584 male and 5878 female samples, and calculated average expression values of coding genes of those proteins in these organs. We found that *C3*, *HP*, *ITIH4*, and *SAA1* genes showed the highest expression levels in the liver, which further confirms that the liver is an important source of those blood proteins. *HP* and *SAA1* mRNAs were expressed in peripheral organs. *ITIH4* mRNA was enriched the liver, blood vessel, cerebellum, and cerebellar hemisphere. *C3* mRNA was widely expressed in all brain regions and most peripheral organs in both male and female, but not in the blood, thus suggesting C3 protein in the blood mainly comes from other organs (especially the liver) rather than blood cells (Figure S2a–c, Supporting Information). HP, ITIH4, and SAA1, as the major acute‐phase biomarker proteins, may rapidly upregulated response to traumatic stimulation such as brain injury.^[^ ^22^, ^23^, ^24^ ^]^ Thus, as expected, they were upregulated in the liver and blood in response to surgical trauma. Although C3 has been reported to be elevated in the brain of AD patients and contribute to synaptic and neuronal loss and cognitive impairment,^[^ ^25^ ^]^ whether peripheral C3 is involved in cognitive impairment and its corresponding pathology is still unclear. Collectively, our results first characterized the proteomic profiles of the liver and blood in aged mice after surgical trauma, and determined four liver‐derived blood proteins, including C3, HP, ITIH4, and SAA1, with the strong changes in response to surgical trauma.

### 2.2 Anesthesia/Surgery Elevates C3 Protein Levels in the Liver, Blood, and Brain of Mice

**Figure 2:**
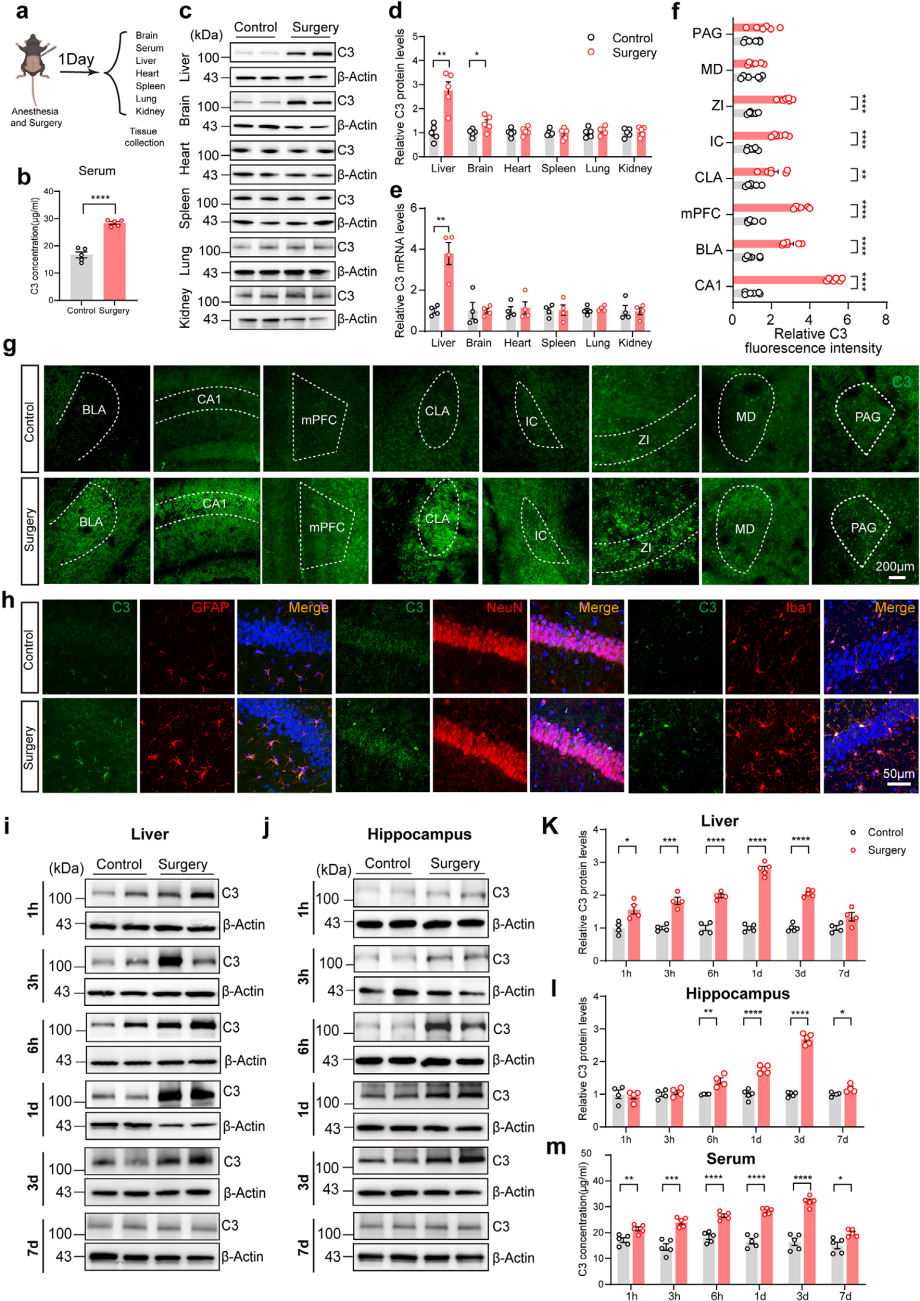
Anesthesia/surgery elevates C3 protein levels in the liver, blood, and brain of aged mice. a) Schematic of the experimental design. b) Serum C3 concentrations in the two groups (n = 5 per group). (c,d) Relative C3 protein levels in the liver, kidney, heart, spleen, lung, and brain of the aged mice after anesthesia/surgery (n = 5 per group). e) Relative C3 mRNA levels in the liver, kidney, heart, spleen, lung, and brain of the aged mice after anesthesia/surgery (n = 4 per group). f) Comparison of fluorescence intensity of C3 between two groups in multiple brain regions (n = 6 per group). g) Representative fluorescence confocal images of the distribution of C3 (green) in the BLA, CA1, mPFC, CLA, IC, ZI, MD, PAG. Scale bars: 200 µm. h) Double immunostaining of C3 (green), GFAP (red), NeuN(red), and Iba11(red) in the hippocampal CA1 region from the control and surgery group (n = 6 per group). Scale bars: 50 µm. i–l) Relative C3 protein levels in the liver and hippocampus between control and surgery groups at 1h, 3h, 6h, 1d, 3d, and 7d after anesthesia/surgery (n = 4–5 per group). m) Serum C3 concentrations between two groups (n = 4 per group) at 1h, 3h, 6h, 3d, and 7d after anesthesia/surgery (n = 5 per group). Statistical significance was determined using two‐tailed unpaired Student's t test or Wilcoxon test. Data are presented as the means ± SEM. *P < 0.05, **P < 0.01, ***P < 0.001, ****P < 0.0001.

To investigate which organs undergo changes in C3 levels as a result of anesthesia/surgery, we employed two well‐established POCD models—laparotomy and internal fixation surgery of tibial fractures, and measured C3 mRNA and protein levels across multiple organs^[^ ^26^, ^27^ ^]^ (**Figure** **2**a). We found that C3 protein levels in the liver, serum and brain synchronously increased 1day after anesthesia/surgery, while the changes in other peripheral organs including the kidney, heart, spleen, and lung were not significant (Figure 2b–d; Figure S3a,b, Supporting Information). The qPCR demonstrated a significant increase in liver *C3* mRNA 1day after anesthesia/surgery, with no notable changes in other organs (Figure 2e; Figure S3c, Supporting Information). These results suggest that the alteration of hippocampal C3 protein is very likely to originate from the periphery.

To investigate the central distribution of C3 protein following anesthesia/surgery, we conducted immunofluorescence staining of C3 in the periaqueductal gray (PAG), mediodorsal thalamus (MD), zona incerta (ZI), insular cortex (IC), claustrum (CLA), medial prefrontal cortex (mPFC), basolateral amygdala (BLA), and hippocampal area cornu ammonis 1 (CA1) between control and surgery mice. We found that C3 levels were significantly increased in 6 brain regions 1d after anesthesia/surgery, with the CA1 showing the most pronounced increase (Figure 2f,g). Furthermore, we co‐stained C3 with markers for astrocytes, microglia, and neurons in the hippocampus, and found that the fluorescence intensity of C3 in astrocytes, microglia, and neurons was elevated after anesthesia/surgery (Figure 2h; Figure S3d, Supporting Information). Given that anesthesia/surgery does not alter *C3* mRNA levels but increases its protein levels in the hippocampus, it is plausible that this elevation  is primarily due to the influx of peripheral C3 into the CNS, although previous studies have reported that C3 can be expressed in astrocytes and microglia.^[^ ^21^, ^28^ ^]^ Considering the hippocampus's well‐recognized function in learning and memory, we focused on this region to further explore the potential sources of C3 and the specific cell types through which it may exert its effects.

To further determine the causal relationship among C3 changes in three organs, we assessed the changed trajectory of C3 at multiple time points after anesthesia/surgery. Western blot showed that C3 levels were significantly increased at 1h, 3h, 6h, 1d, and 3d in the liver of surgery mice compared to that of control mice, and returned to normal levels at 7d. Interestingly, hippocampal C3 levels exhibited no obvious changes until 6h after anesthesia/surgery (Figure 2i–l). In addition, the changes of serum C3 levels were similar to the liver after anesthesia/surgery (Figure 2m). At the transcriptional levels, *C3* mRNA was significantly increased in the liver at 1d and 3d, and returned to normal levels at 7d after anesthesia/surgery (Figure 2e; Figure S3e, Supporting Information). However, mRNA levels of *C3* gene in the hippocampus showed no significant difference between the two groups at 3d and 7d (Figure S3f, Supporting Information). These results demonstrate that the increase in C3 in the liver and serum induced by anesthesia/surgery occurs earlier than that in the hippocampus, suggesting that the changes in C3 observed in the serum and hippocampus may primarily originate from the liver.

Given our previous findings that anesthesia/surgery can cause transcriptional changes by affecting methylation patterns around promoters of genes,^[^ ^29^, ^30^ ^]^ we speculated whether the upregulation of C3 in the liver could be attributable to anesthesia/surgery‐induced epigenetic modification dysregulation. To verify our conjecture, we performed Bulk‐RNA sequencing and targeted bisulfite sequencing in the paired liver samples between surgery and control mice. We found that methylation levels of 9 CpG sites in surgery mice, including 149, 389, 501, 540, 684, 773, 947, 956, and 1712 that represents the distance between methylation entity and transcription start site (TSS) in *C3* gene promoter, were significantly decreased compared to control mice (Figure S3g; Table S4, Supporting Information); conversely, the *C3* transcription levels in surgery mice were significantly higher than those in control mice (Figure S3h; Table S5, Supporting Information). In addition, Spearman correlation analysis showed that methylation levels of these sites were significantly negatively correlated with *C3* mRNA levels (Figure S3I, Supporting Information). Collectively, these findings reveal that anesthesia/surgery‐induced C3 upregulation in the liver may be associated with hypomethylation of *C3* gene promoter.

### 2.3 Liver‐derived C3 Enters the Brain Under the Premise of Anesthesia/Surgery‐Induced BBB Disruption

**Figure 3:**
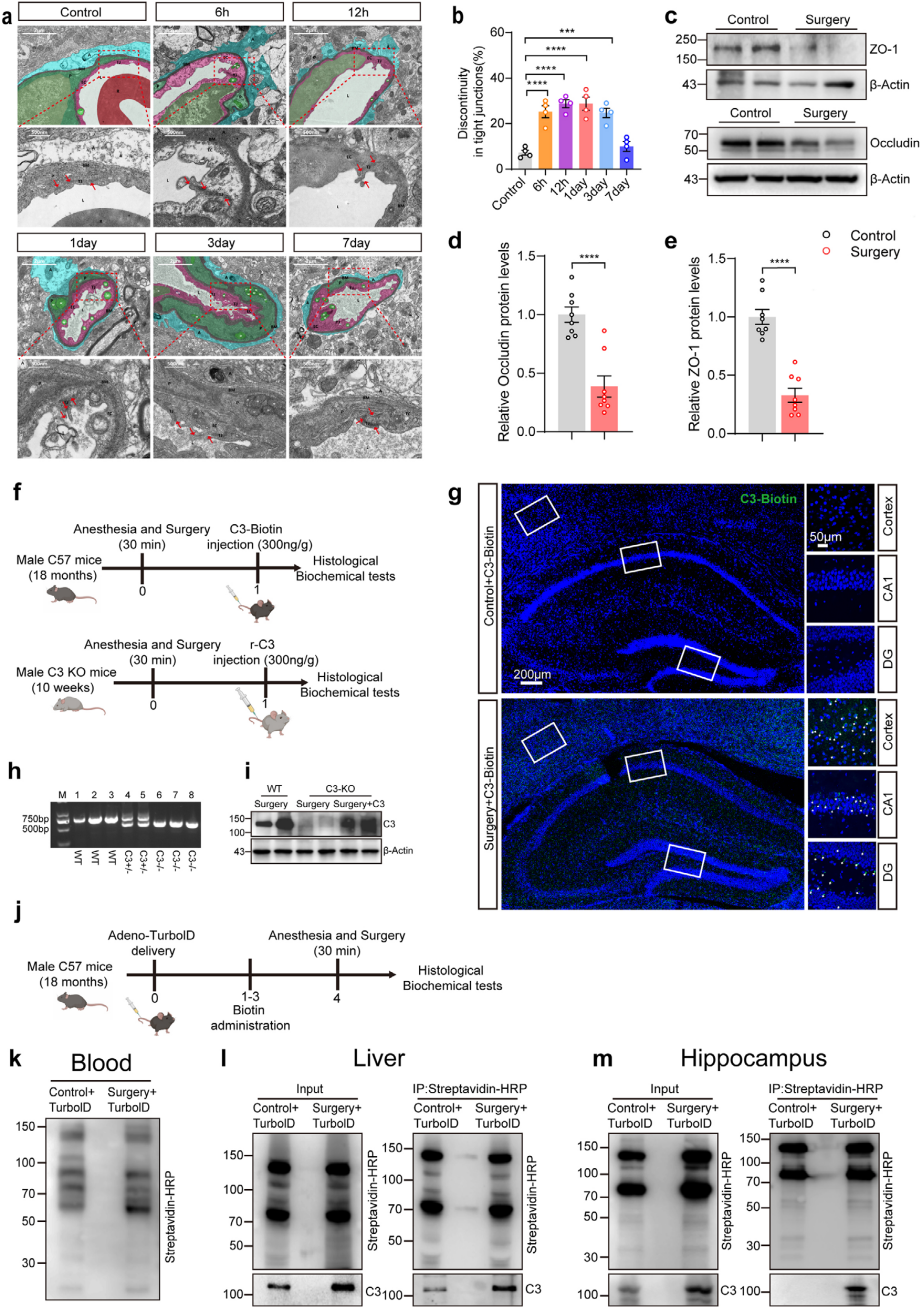
Peripheral C3 enters the brain under the premise of anesthesia/surgery‐induced BBB disruption. a)Transmission electron microscopy (TEM) image of the BBB in mice at 6h, 3d, and 7d after anesthesia/surgery. The red box highlights typical tight junction (TJ) gaps. 1. Control group: Intact tight junction (TJs, red arrows in boxes), quiescent endothelial cells (ECs; purple; smooth surface, uniform cytosol), continuous basement membrane (BM, grey), adherent astrocyte end‐feet (blue). 2. Anesthesia/surgery group: Pathological TJ discontinuities (red arrows), EC microvilli (purple), focal BM thickening (grey), detached astrocyte end‐feet (blue gaps), and luminal collapse (L). b) Statistical analysis of (a) showing the fraction of TJ strands with gaps (n = 4 per group). c–e) Representative image and statistical analysis of Western blot for the expression of ZO‐1 and Occludin in control and surgery mice (n = 8 per group). (f) Schematic diagram of C3 recombinant protein injection via the tail vein. g) Immunofluorescent staining of peripheral biotinylated C3 in the DG, CA1, and cortex of the 18‐month‐old mice with/without anesthesia/surgery. Samples were collected 1d after anesthesia/surgery and 6h after C3 recombinant injection. DG, dentate gyrus. h) PCR genotyping showed that homozygous (C3^−/−^) (lanes 6, 7, and 8) mice were successfully generated. The lanes 2 and 3 represented wild‐type mice (WT). The lanes 4 and 5 represented heterozygous (C3^+/−^) mice. i) Western blot analysis of C3 levels in the hippocampus of C3‐KO mice after tail vein injection of r‐C3 protein. j) Schematic diagram of adenoviral expression of Sec61b‐V5‐TurboID and biotin labeling in the mouse liver tissues. k) Streptavidin‐HRP detection of biotinylated proteins in the blood from Sec61b‐V5‐TurboID adenovirus (AdV) transduced mice. l,m) Immunoprecipitation of hepatic biotinylated C3 proteins detected in the liver and hippocampus 1d after anesthesia/surgery. Laparotomy was used as a surgical method under anesthesia in mice. Statistical significance was determined using one‐way ANOVA with the Dunnett's post‐hoc test. Data are presented as the means ± SEM. ***P < 0.001, ****P < 0.0001. A scale bar was shown in the figures.

Blood‐brain barrier (BBB) is a natural protective membrane that prevents the entry of blood‐derived products, pathogens, and cells into the brain.^[^ ^31^ ^]^ BBB disruption is linked to aging, neurodegenerative disorders, and cognitive impairment.^[^ ^32^ ^]^ To evaluate the effects of anesthesia/surgery on the ultrastructure of the BBB, we examined the hippocampal endothelial cell (EC) junctions in the aged mice using transmission electron microscopy (TEM). Compared to control mice, the surgery group exhibited discernible discontinuities within the BBB structure (**Figure** **3**a). Furthermore, we observed a time‐dependent impairment of tight junctions (TJs): initial signs of TJ damage emerged at 6 h post‐surgery, peaked in severity at 3 days, and showed signs of recovery at 7 days (Figure 3b). This structural impairment was further validated at the molecular level by western blot (WB) analysis, which revealed an average 50% decrease in the hippocampal expression of key TJ‐associated proteins, including ZO‐1 and Occludin 1d after anesthesia/surgery (Figure 3c–e). These results confirm that anesthesia/surgery can induce BBB disruption.

Considering that C3 may exceed the molecular weight normally allowed to pass through the BBB, we speculated that C3 may enter the brain when anesthesia/surgery resulted in the BBB breakdown. To test this theory, we labeled recombinant C3 protein with biotin (≈0.45 kDa) and administered it via tail vein injection in 18‐month‐old WT mice and 10‐week‐old homozygous C3 knockout(C3^−/−^) mice with or without anesthesia/surgery (Figure 3f). The results of the immunofluorescence assays revealed that 6 hours after the administration of recombinant C3, biotinylated C3 was exclusively detected in the cortex and hippocampus of surgery mice, while it was undetectable in control mice (Figure 3g). Additionally, WB analysis demonstrated a significant presence of C3 in the hippocampus of both WT and C3KO mice that received recombinant C3 protein following anesthesia/surgery, unlike in mice that did not undergo anesthesia/surgery (Figure 3h,i; Figure S4a,b, Supporting Information).

Finally, to investigate whether hepatogenous C3 protein can enter the brain, we employed the iSLET (in situ Secretory protein Labeling via ER‐anchored TurboID) system, a validated technique that enables liver‐specific labeling of secretory proteins through ER‐anchored proximity biotinylation (Experimental Section).^[^ ^33^ ^]^ Briefly, we administered Sec61β‐TurboID‐expressing adenovirus via tail vein injection followed by biotin treatment to selectively label liver‐derived secretory proteins in control and surgery mice (Figure 3j). Immunofluorescence in the liver tissue showed that Sec61β‐TurboID was robustly expressed, and Streptavidin‐HRP detection of liver proteins revealed that those liver proteins are efficiently biotinylated (Figure S4c, Supporting Information). Moreover, we could unambiguously detect Sec61b‐TurboID‐dependent biotinylated liver secretory proteins in the blood among the two groups (Figure 3k).

Critically, our immunoprecipitation (IP) results further indicated that biotinylated liver‐derived C3 is elevated in the liver of surgery mice compared to control mice (Figure 3l; Figure S4d, Supporting Information). Additionally, biotinylated liver‐derived C3 was exclusively detected in the hippocampus of the surgery mice, but not in the control mice (Figure 3m; Figure S4e, Supporting Information). These results reveal that hepatogenous C3 protein can enter the hippocampus through the circulatory system.

Taken together, these findings demonstrate that large amounts of peripheral C3 enters the CNS only when the BBB is damaged. Anesthesia/surgery not only induces BBB breakdown but also results in hepatogenic C3 protein elevations in the blood. These dual effects of surgical intervention ultimately facilitate the influx of C3 into the brain.

### 2.4 Knockdown of Liver‐derived C3 Alleviates Anesthesia/Surgery‐Induced Cognitive Impairment

**Figure 4:**
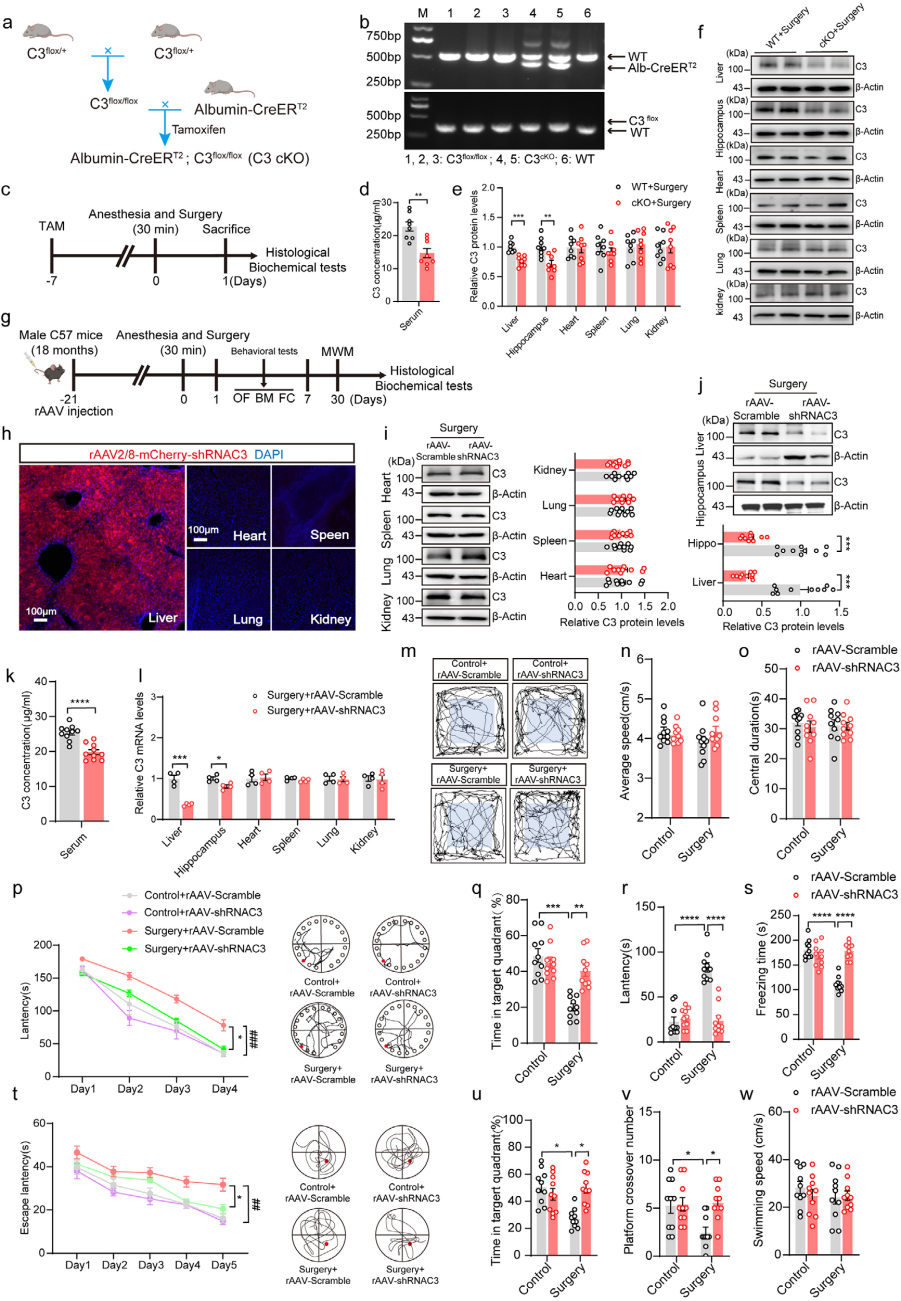
Knockdown of liver‐derived C3 alleviates anesthesia/surgery‐induced cognitive impairment. a) Schematic of the generation of cKO mice with liver‐specific deletion of C3 (*Albumin‐CreER^T2^*; *C3^f/f^*). b) Validation of cKO mice via genotyping. c) Schematic of the experimental design of WT and cKO mice. d–f) Relative C3 protein levels in the serum, liver, hippocampus, kidney, heart, spleen, and lung between WT and cKO mice after anesthesia/surgery (n = 8 per group). g) Schematic of the experimental design of liver‐specific C3 knockdown. h) rAAV2/8‐mCherry‐shRNA C3 was specifically expressed in the liver 3 weeks after tail‐vein injection. Scale bar: 100 µm. i) Relative C3 protein levels in the kidney, heart, spleen, and lung of mice with or without knockdown of C3 (n = 10 per group). j) Relative C3 protein levels in the liver and hippocampus of mice with or without knockdown of C3 (n = 10 per group). k) Serum C3 concentrations in mice with or without knockdown of C3 (n = 10 per group). l) Relative C3 mRNA levels in the liver, hippocampus, kidney, heart, spleen, and lung of mice with or without knockdown of C3 (n = 4 per group). m) Representative video tracking images of open field test between four groups. The average speed n) and central duration o) of mice in the open field tests (n = 10 per group). p–r) Results of the Barnes maze tests of mice (n = 10 per group). Mean latency of mice in the training phase of 4 consecutive days (p). Time (%) spent in the target quadrant of mice in the testing phase (q). The latency of first appeared at the target (r). s) Freezing time of mice in the fear conditioning test (n = 10 per group). (t‐w) Results of the Morris water maze test (n = 10 per group). The latency to find the platform during the training stage between groups t). The time spent in the target quadrant u), crossover number v), and swimming speed w) in the target quadrant during the MWM testing stage between groups (n  =  10 per group). Laparotomy was used as a surgical method under anesthesia in mice. Two‐way repeated‐measures ANOVA with Bonferroni's post hoc test for time × group comparisons. Student's t test or Wilcoxon test was used to determine the statistical significance of differences between two groups. Statistical significance of the four groups was determined using one‐way ANOVA with Tukey's post‐hoc test or Kruskal‐Wallis Test & Dunn's test. Data are presented as the means ± SEM. *P < 0.05, **P or ^##^ *P* < 0.01, ***P or ^###^ *P* < 0.001, ****P < 0.0001. A scale bar was shown in the figure.

To further determine whether liver‐derived C3 regulates C3 levels in the CNS, we first performed experiments using *Albumin‐creER* ^T2^; *C3* ^loxp/loxp^ double‐transgenic mice, in which hepatogenous C3 was deleted following a 7‐d tamoxifen (TAM) injection (henceforward referred to as cKO mice) (**Figure** **4**a,b). The C3 levels from WT and cKO mice were measured 1d after anesthesia/surgery (Figure 4c). We found that C3 levels of the liver, serum, and hippocampus in cKO mice were consistently decreased compared to WT mice, whereas C3 levels in other peripheral organs were unaffected by hepatogenous C3 ablation (Figure 4d–f). Additionally, immunostaining revealed that C3 fluorescence density was significantly reduced in astrocytes, microglia, and neurons in the hippocampus of cKO mice (Figure S5a–f, Supporting Information). In addition, we specifically knocked down C3 in the liver of 18‐month‐old mice via intravenous injection with recombinant adeno‐associated virus (rAAV) 2/8‐mCherry‐shRNAC3 (Figure 4g). Immunofluorescence detected mCherry^+ cells in the liver but not in other organs after 3 weeks (Figure 4h), suggesting that the virus specifically transfected the liver. We found that rAAV‐shRNAC3 injection significantly reduced C3 levels in the liver, serum, and hippocampus (Figure 4i–k), and rAAV‐shRNAC3 injection only inhibited the transcription of C3 in the liver (Figure 4l). Together, these findings demonstrate that the liver plays a regulatory role in controlling hippocampal C3 levels following anesthesia/surgery.

We then assessed the effect of liver‐specific C3 knockdown on the anesthesia/surgery‐induced neurological deficits using behavioral tests. In open field test (OFT), four groups showed no difference (Figure 4m–o). Intriguingly, we found that rAAV‐shRNAC3 injection alleviated the anesthesia/surgery‐induced cognitive impairment in the Barens Maze and fear conditioning test (FC) (Figure 4p–s). Moreover, rAAV‐shRNAC3 mice showed significantly improved cognitive performance compared to rAAV‐Scramble mice in the Morris water maze (MWM) (Figure 4t–w). Additionally, 18‐month‐old C3KO mice that underwent anesthesia/surgery showed a better learning and memory performance in the Barens Maze, FC, and MWM compared to WT mice (Figure S6, Supporting Information). Similarly, adult 5×FAD mice injected with rAAV‐shRNAC3 also showed significant cognitive improvement (Figure S7, Supporting Information). Collectively, these results demonstrate that inhibition of liver‐derived C3 can ameliorate cognitive impairment induced by anesthesia/surgery, displaying that hepatogenous C3 contributes to the impairment of cognitive function.

Given the essential role of C3 in the immune system, we further examined whether liver‐specific C3 knockdown could affect the histological integrity of peripheral organs or alter immune‐related hematological parameters. No significant histopathological alterations were observed in the heart, liver, spleen, lung, or kidney after liver‐specific C3 knockdown, as assessed by histological observation (Figure S8a, Supporting Information). Strikingly, liver‐specific C3 knockdown significantly attenuated the anesthesia/surgery‐induced liver function injury (Figure S8b, Supporting Information). Indicators of renal function, containing blood urea nitrogen (BUN), creatinine (CREA), and uric acid (UA) were observed no difference among four groups (Figure S8c, Supporting Information). The analysis of body weight and routine blood parameters, including white blood cell (WBC) count, lymphocyte count, and neutrophil count, showed no statisticaldifference between four groups (Figure S9, Supporting Information). The results imply that inhibiting liver‐derived C3 does not adversely affect peripheral organs, as supported by comprehensive safety evaluations, and underscore the systemic safety of liver‐specific C3 inhibition. Instead, it may avoid the damage to liver function caused by anesthesia/surgery.

### 2.5 Knockdown of Hepatogenous C3 Rescues Anesthesia/Surgery‐Induced Synaptic Dysfunction

**Figure 5:**
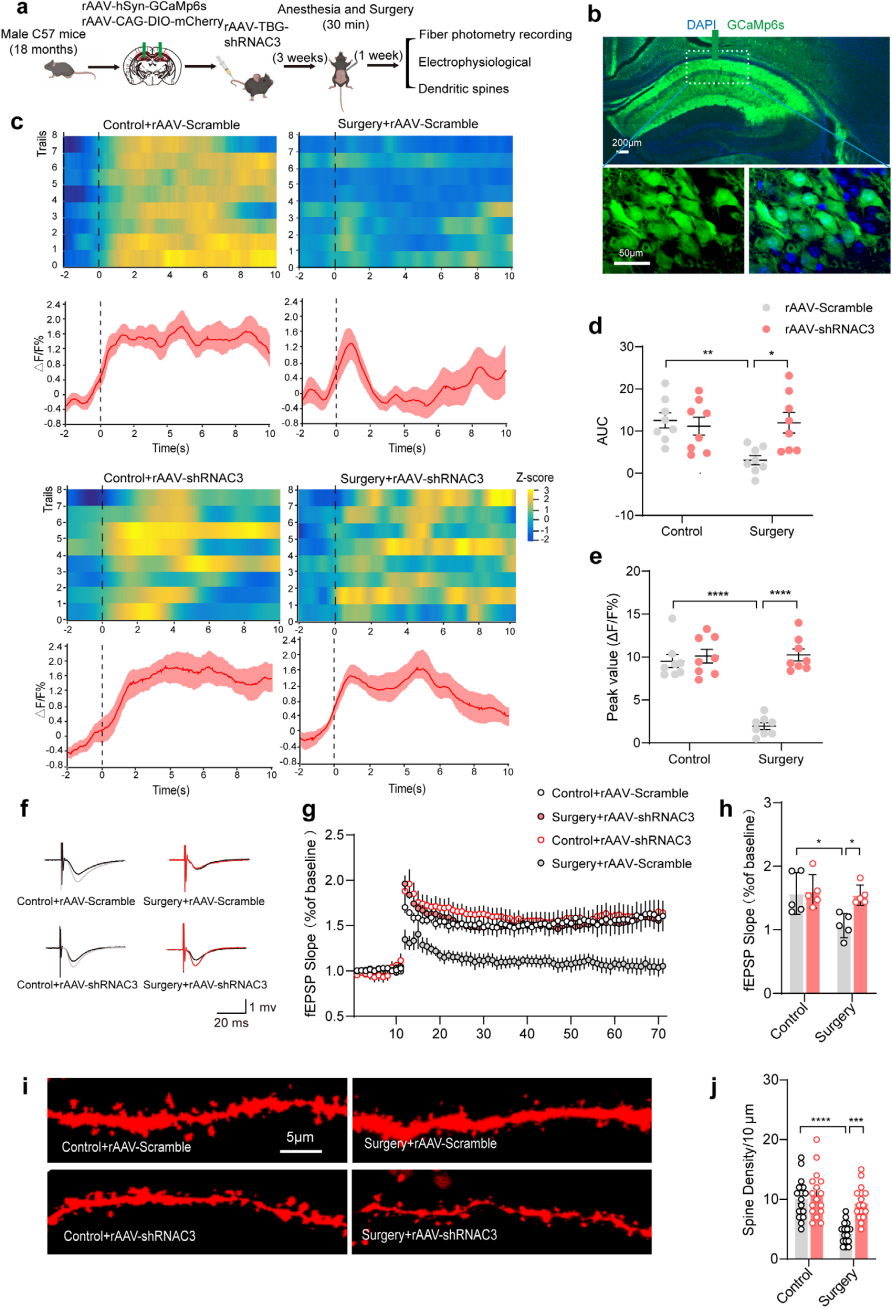
Knockdown of hepatogenic C3 rescues anesthesia/surgery‐induced synaptic dysfunction. a) Schematic diagram of experimental design. b) Representative images of GCaMP6s expression in the hippocampus of mice. c) Heatmap showing calcium activities at the onset of freezing epoch of four groups in the fear conditioning test. Calcium transients were normalized and shown as Z‐score, and each recording window covered 2 s before and 10 s after the transition (n = 8 per group). d,e) Area under the curve and calcium peak of calcium activities at the onset of freezing epoch (n = 8 per group). f–h) Normalized the fEPSP slope and quantitative analysis in the hippocampus of four groups (n  =  5 per group). i,j) Representative high‐magnification confocal images of dendritic spines and quantification of spine density in the hippocampus (n = 16 dendrites from 3 mice per group). Laparotomy was used as a surgical method under anesthesia in mice. Statistical significance of the four groups was determined using one‐way ANOVA with Tukey's post‐hoc test or Kruskal‐Wallis Test & Dunn's test. Data are presented as the means ± SEM. *P < 0.05, **P < 0.01, ***P < 0.001, ****P < 0.0001. A Scale bar was shown in the figure.

Synaptic integrity, activity, and plasticity are essential for learning and memory.^[^ ^34^ ^]^ Previous studies reported that C3 in the brain disrupts dendritic morphology and neuronal network function,^[^ ^19^ ^]^ and C3KO mice are protected against hippocampal synaptic loss and cognitive decline during normal aging.^[^ ^25^ ^]^ These evidences provided us with a hypothesis that anesthesia/surgery could lead to an increase in hippocampal hepatogenic C3, which impairs synapse when the BBB is damaged by anesthesia/surgery. To test our hypothesis, we used fiber photometry recording, electrophysiology, and immunofluorescence to assess synaptic structure and function in the hippocampus of four groups (**Figure** **5**a). First, we performed calcium photometry to monitor the hippocampal neuronal activity among four groups. The genetic manipulation of hepatogenic C3 was combined with GCaMP6s infection to track the in vivo neural activity of the hippocampus, from which Ca2+ activity of GCaMP6s‐expressing neurons was presented using a miniaturized microscope during a fear conditioning test (Figure 5b). We found that the calcium activity of neurons in surgery mice injected with rAAV‐Scramble was decreased during the test compared to control mice injected with rAAV‐Scramble, while calcium activity in surgery mice injected with rAAV‐shRNAC3 was similar to that of the control mice injected with rAAV‐Scramble, with a significant enhancement compared to surgery mice injected with rAAV‐Scramble (Figure 5c–e). We then assessed hippocampal synaptic plasticity utilizing electrophysiology. The poorer induction and maintenance of long‐term potentiation (LTP) and a lower level of average fEPSPs were observed in the hippocampus of surgery mice injected with rAAV‐Scramble than that of other three groups (Figure 5f–h). Finally, synaptic morphology and integrity in the hippocampus of four groups were evaluated. We injected rAAV‐CAG‐DIO‐mCherry into the hippocampus, targeting a sparse subset of hippocampal neurons, to investigate synaptic modifications. Immunofluorescence further exhibited that dendritic spine density in surgery mice injected with rAAV‐Scramble was notably reduced compared with control mice injected with rAAV‐Scramble, while surgery mice injected with rAAV‐shRNAC3 had an increased dendritic spine density relative to surgery mice injected with rAAV‐Scramble, with a similar synaptic morphology to control mice injected with rAAV‐Scramble (Figure 5i,j). The above results support our hypothesis that when the BBB is damaged by anesthesia/surgery, liver‐derived C3 can enter the CNS and injures synaptic structure and function. Inhibiting its source could rescue anesthesia/surgery‐induced synaptic disorder including impaired neural activity and damaged synaptic integrity and plasticity.

### 2.6 Inhibition of Hepatogenic C3 Attenuates Anesthesia/Surgery‐Induced C3aR Activation and Microglial Phagocytosis

**Figure 6:**
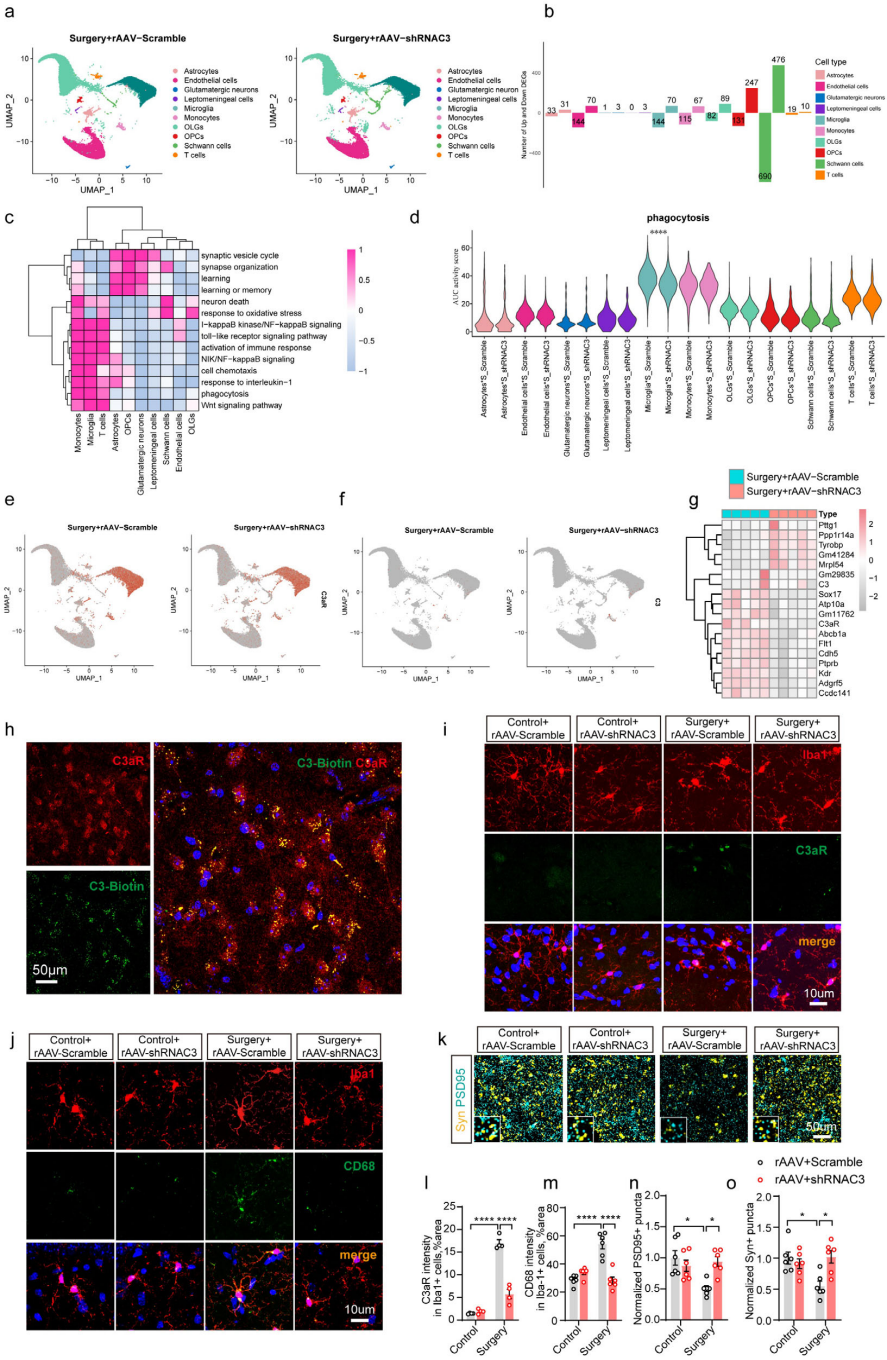
Inhibition of hepatogenic C3 attenuates anesthesia/surgery‐induced C3aR activation and microglial phagocytosis. a–f) The results of single‐cell sequencing (scRNA‐Seq) in the hippocampus between anesthesia/surgery mice injected with rAAV‐Scramble and surgery mice injected with rAAV‐shRNAC3 (n  = 3 per group). (a) Identification of 10 hippocampal cell types in two groups of mice (67617 cells). (b) The numbers of upregulated and downregulated differentially expressed genes (DEGs) in hippocampal cell types between two groups of mice. The |log2 (fold change) | > 0.25 and Benjamini‐Hochberg adjusted *P* value < 0.05 was regarded as the cutoff. (c) Single‐cell biological function analysis using the scMetabolism package based on AUCell activity scores of the DEGs. (d) Differential biological function analysis for phagocytosis based on the single‐cell AUCell scores within each cell type. (e,f) The UMAP visualization of *C3aR* (e) and *C3* (f) genes in hippocampal cells from two groups of mice. g) Heatmap showing the downregulated and upregulated DEGs in the hippocampus between two groups of mice by Bulk‐RNA sequencing (n  =  5 per group). Differential gene analysis was performed using the edgeR package. The |log (fold change) | > 0.58 and Benjamini‐Hochberg adjusted *P* value < 0.05 were applied as the cutoff for screening DEGs. h) Intravenous injection of biotinylated C3 resulted in specific protein binding onto hippocampal cells expressing C3aR in mice. i,l) Representative co‐immunostaining and quantification of C3aR and Iba1 in the hippocampus of four groups of mice (n  =  4 per group). j,m) CD68 and Iba1 co‐immunostaining and quantification in the hippocampus of four groups of mice (n  =  4 per group). k) Confocal images of pre‐ and postsynaptic terminals labeled against synaptophysin and PSD95 in the hippocampus of four groups of mice, respectively. n,o) Quantification of synaptic puncta in the hippocampus of four groups of mice (n = 6 per group). Laparotomy was used as a surgical method under anesthesia in mice. Student's t test was used to determine the statistical significance of differences between two groups. Statistical significance of the four groups was determined utilizing one‐way ANOVA with Tukey's post‐hoc test. Data are presented as the means ± SEM. *P < 0.05, **P < 0.01, ***P < 0.001, ****P < 0.0001. A scale bar was shown in the figure.

To determine how downstream signaling from hepatogenic C3 in the hippocampus affects cognitive function, we conducted single‐cell RNA sequencing (scRNA‐seq) and bulk‐RNA sequencing on hippocampal samples from mice injected with rAAV‐Scramble and those injected with rAAV‐shRNAC3. Initial analysis identified 34 cell clusters across two groups (Figure S10a, Supporting Information). After quality control as well as alignment with known cell markers (Figure S10b, Supporting Information), these clusters were categorized into 10 cell types, including microglia, astrocytes, monocytes, oligodendrocytes (OLGs), oligodendrocyte precursor cells (OPCs), leptomeningeal cells, glutamatergic neurons, Schwann cells, endothelial cells, and T cells (**Figure** **6**a). No significant differences were observed in the proportions of hippocampal cells between the two groups (Figure S10c, Supporting Information).

Differential expression analysis identified distinct numbers of upregulated and downregulated differentially expressed genes (DEGs), demonstrating that the inhibition of hepatogenic C3 induces specific gene expression changes in hippocampal cells (Figure 6b; Figure S11, and Table S6, Supporting Information). To further elucidate the functions of these DEGs, we conducted a single‐cell biological function analysis using the scMetabolism package based on AUCell activity scores of DEGs, which corresponded to multiple cognition‐related biological processes and pathways (see Experimental Section). Our results indicated that microglia, monocytes, and T cells were primarily involved in pathways related to neuronal death, phagocytosis, phagocytic engulfment, and neuroinflammation. In contrast, astrocytes, OPCs, and glutamatergic neurons were implicated in processes related to learning, memory, synaptic vesicle cycling, and synapse organization (Figure 6c). Gene Ontology (GO) enrichment and KEGG pathway enrichment analyses revealed that DEGs associated with neuronal death, phagocytosis, phagocytic engulfment, and neuroinflammation‐related pathways were widely downregulated across hippocampal cells (Figure S10d; Table S7, Supporting Information). Conversely, DEGs linked to learning or memory, synaptic vesicle cycling, and synapse organization were broadly upregulated in astrocytes, OLGs, OPCs, and Schwann cells (Figure S10e; Table S7, Supporting Information). Additionally, differential biological function analysis based on the aforementioned single‐cell AUCell scores within each cell type demonstrated that the activities related to phagocytosis, phagocytic engulfment, NIK/NF‐kappaB signaling, activation of immune response, interleukin 1‐mediated signaling, and response to interleukin‐1 were specifically reduced in microglia from mice injected with rAAV‐shRNAC3 compared to those injected with rAAV‐Scramble (Figure 6d; Figure S10f–h and Table S8, Supporting Information). Notably, *C3aR* gene enriched in phagocytosis was found to be mainly expressed in microglia, which is a critical driver for cognitive impairment,^[^ ^35^ ^]^ and was downregulated in microglia, astrocytes, and OPCs from surgery mice injected with rAAV‐shRNAC3 (Figure 6e). Interestingly, our results also indicated that *C3* gene was expressed at a very low level in an extremely small number of microglia and monocytes (Figure 6f). Furthermore, bulk RNA sequencing confirmed that *C3aR* mRNA levels were reduced in the hippocampus of surgery mice injected with rAAV‐shRNAC3, whereas *C3* mRNA showed no significant difference between two groups (Figure 6g; Table S9, Supporting Information). Moreover, CR3 and C5aR, the critical complement receptors implicated in neuroinflammation and complement activation,^[^ ^18^, ^36^ ^]^ had no significant changes following inhibition of liver‐specific C3 in the hippocampus (Figure S12a–d, Supporting Information). Collectively, these findings indicate that the inhibition of hepatogenous C3 does not affect the expression levels of the *C3* gene in the hippocampus of mice, but selectively leads to a decrease in C3aR expression in microglia, thereby may reduce C3aR‐mediated phagocytic activation.

A previous study reported that the reduction of glial C3 production inhibits C3‐C3aR signaling‐mediated glial activation, potentially preventing synaptic loss, synaptic phagocytosis, and cognitive impairment.^[^ ^21^ ^]^ The overactivated microglial phagocytosis of neurons and synapses leads to multiple neurodegenerative diseases and cognitive impairment.^[^ ^37^ ^]^ Given our results and the reported findings, we hypothesized that anesthesia/surgery could activate hippocampal C3‐C3aR signaling‐mediated microglial phagocytosis of synapses. To test this, we injected biotinylated C3 protein into the aged mice via the tail vein after anesthesia/surgery. Immunostaining demonstrated that biotinylated C3 colocalized with C3aR on hippocampal cells because peripheral C3 produced fluorescent signals on hippocampal cells expressing C3aR (Figure 6h). Moreover, C3aR levels were demonstrated to be significantly upregulated in microglia after anesthesia/surgery (Figure 6i,l), suggesting a crucial role in the microglial response to anesthesia/surgery. We then examined the expression of the phagocytosis markers CD68 and Iba‐1, and the results revealed a significant increase in the number of CD68^+^ lysosomes within microglia following anesthesia/surgery (Figure 6j,m). Confocal images of Synaptophysin (Syn)^+^ presynapses and PSD95^+^ postsynapses were examined to determine the quantity of synapses, which play the central roles in learning and memory.^[^ ^38^, ^39^ ^]^ The analysis demonstrated a reduction in synaptic puncta density after anesthesia/surgery (Figure 6k,n,o). Interestingly, the inhibition of hepatogenous C3 can reverse the upregulation of C3aR expression, the abnormal activation of microglia, and the loss of synapses induced by anesthesia/surgery (Figure 6i–o).

Previous studies have suggested that cancer‐cell‐derived C3 can disrupt the blood‐CSF barrier by activating C3aR in the choroid plexus epithelium.^[^ ^40^ ^]^ To determine whether C3aR signaling contributes to BBB disruption following anesthesia/surgery, we investigated whether C3aR signaling is required for peripheral C3 to cross the BBB. We injected biotinylated C3 protein intravenously (IV) into surgery mice treated with C3aR antagonist SB290157 and surgery mice treated with dimethyl sulfoxide (vehicle) 1d after anesthesia/surgery. The results revealed that biotin‐C3 was observed within the hippocampus of both SB290157‐treated and vehicle‐treated mice after anesthesia/surgery (Figure S13a, Supporting Information), suggesting that blocking C3aR signaling does not prevent peripherally administered C3 from entering the brain parenchyma following anesthesia/surgery. This provides direct evidence that C3 entry across the disrupted BBB is independent of C3aR activation. TEM analysis indicated that the extent of BBB damage observed in SB290157‐treated mice at 6 h post‐surgery was comparable to that in vehicle‐treated surgery mice (Figure S13b, Supporting Information), indicating that hippocampal C3aR intervention does not prevent anesthesia/surgery‐induced BBB disruption.

Together, the above findings demonstrate that anesthesia/surgery activates C3‐C3aR signaling through the entry of peripheral C3 into the brain, which promotes abnormal phagocytosis of synapses by microglia. In contrast, the inhibition of liver‐specific C3 can mitigate these processes.

### 2.7 Perioperative Serum C3 Protein Changes are Associated with Postoperative Cognitive Impairment

**Figure 7:**
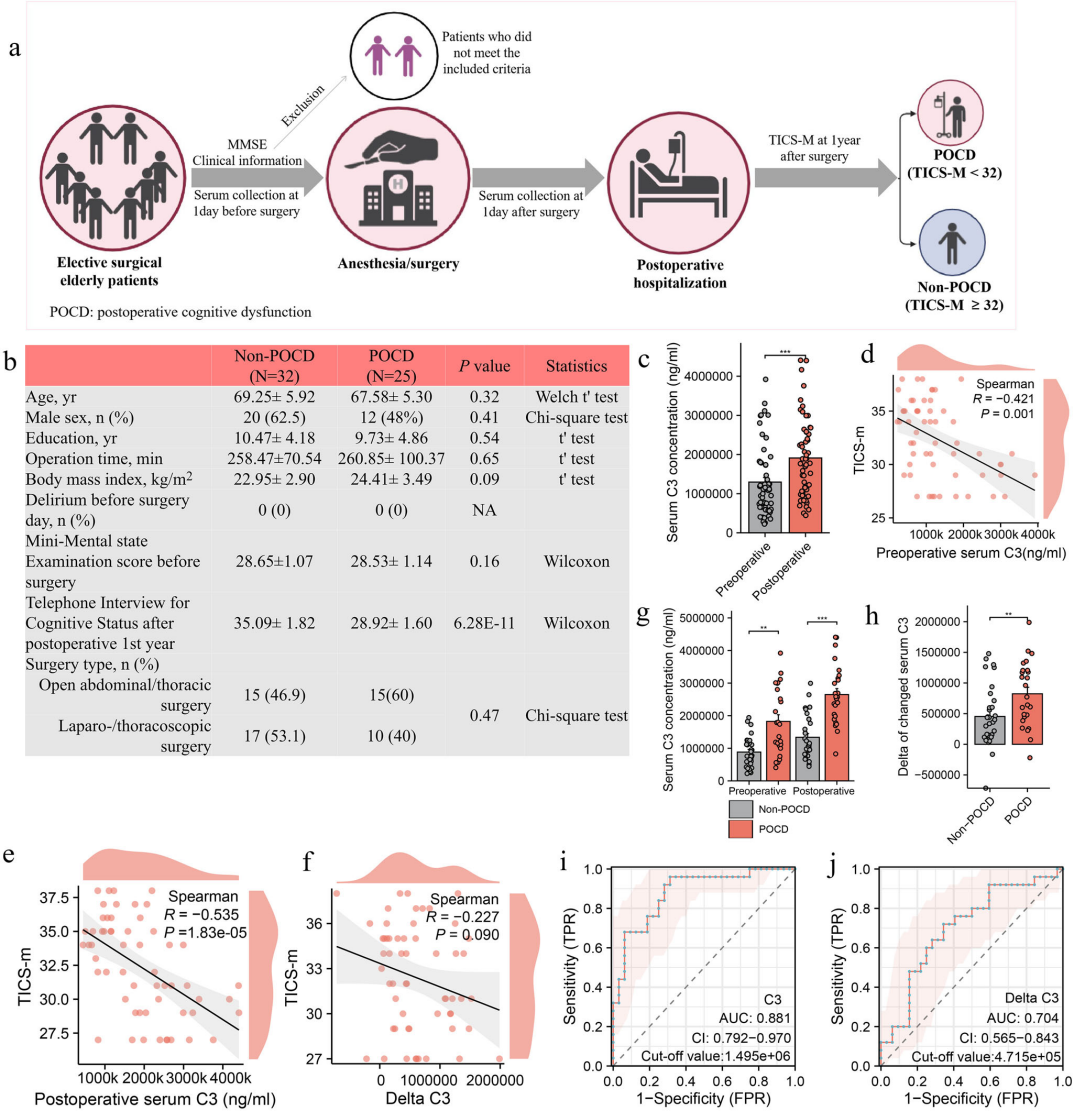
Perioperative serum C3 protein alterations are linked to postoperative cognitive impairment. a) Flow diagram of observational study. b) Clinical information of 57 surgical patients with general anesthesia including 32 non‐POCD and 25 POCD. c) Comparations of serum C3 concentrations in 57 surgical patients. d) Correlation of preoperative serum C3 protein concentrations 1 day before surgery with TICS‐M (cognitive index score) 1 year after surgery. e) Association between postoperative serum C3 concentrations 1 day after surgery and TICS‐M 1 year after surgery. f) Relationship of the delta value of postoperative and preoperative changes in serum C3 concentrations with TICS‐M. g) Comparations of postoperative and preoperative serum C3 concentrations between non‐POCD and POCD patients. h) Comparations of delta value of postoperative and preoperative changes in serum C3 concentrations between non‐POCD and POCD patients. i,j) Receiver operating characteristic (ROC) curve analysis for postoperative POCD susceptibility based on serum C3 levels at 1 day after surgery and delta value of postoperative and preoperative changes in C3. The Wilcoxon test was used for comparisons between the two groups. Kruskal‐Wallis Test & Dunn's test was used for multiple comparisons between the 4 groups. Spearman analysis was performed on two groups of continuity variables. *P < 0.05, ** *P* < 0.01, ***P < 0.001. Error bars represent SEM.

We next investigated the impact of anesthesia/surgery on serum C3 of elderly surgical patients with or without POCD, and systematically evaluated the associations of perioperative C3 changes with postoperative cognitive impairment. The inclusion and exclusion criteria for elective surgical elderly patients and the diagnosis of POCD are shown in **Figure** **7**a and Experimental Section. Clinical information of surgical patients included in this study is summarized in Figure 7b. A total of 57 surgical patients consisting of 32 non‐POCD (15 open abdominal/thoracic surgery and 17 laparo‐/thoracoscopic surgery) and 25 POCD (15 open abdominal/thoracic surgery and 10 laparo‐/thoracoscopic surgery) patients with general anesthesia were included. All subjects had no cognitive impairment (Mini‐Mental State Examination score > 27) before surgery and did not develop delirium within 1 week after surgery. There were no obvious differences in age, sex, education, operation time, body mass index, pathologically diagnosed cancer types between non‐POCD and POCD patients. The ELISA results indicated that serum C3 concentrations were significantly higher in  postoperative non‐POCD and POCD patients compared to their preoperative counterparts in both groups (Figure 7c), indicating anesthesia/surgery can enhance serum C3 levels. Spearman correlation analysis exhibited that preoperative and postoperative C3 levels of all surgical patients had an obviously negative correlation with cognitive index (Telephone Interview for Cognitive Status score), respectively (‐0.53 ≤ R < ‐0.42, *P* < 0.05) (Figure 7d,e). Moreover, the delta value of postoperative and preoperative changes in serum C3 of those patients also showed a negative association with cognitive index (R = ‐0.23, *P* = 0.09) (Figure 7f). Those results show that alterations of serum C3 of surgical patients are linked to poor postoperative cognitive function.

Differential analysis further exhibited that preoperative and postoperative serum C3 concentrations of POCD patients were higher than those in non‐POCD patients, respectively (Figure 7g). In addition, the delta value of preoperative and postoperative changes in serum C3 was also increased in POCD patients compared to non‐POCD patients (Figure 7h). Finally, receiver operating characteristic (ROC) curve analysis indicated that serum C3 levels at 1 day after surgery and the delta value of preoperative and postoperative changes in C3 had a relatively good predictive ability for postoperative POCD, and their corresponding cut‐off value was 1.495e+06 (ng/mL) and 4.715e+05 (delta), respectively (Figure 7i,j). Collectively, these findings demonstrate that perioperative serum C3 changes are associated with postoperative cognitive impairment, with the potential to predict POCD.

## 3 Discussion

Postoperative cognitive dysfunction (POCD) is a common postoperative cognitive impairment mainly caused by anesthesia/surgery in the elderly.^[^ ^3^, ^4^ ^]^ Currently, researches on its associated pathogenic mechanisms mainly focus on the CNS. Blood proteins actively released into the blood by organs have an important impacts on the onset of neurological diseases.^[^ ^9^, ^11^, ^14^, ^16^, ^17^ ^]^ In this study, we aimed to explore whether anesthesia/surgery‐induced peripheral blood protein disorders mediate the susceptibility to POCD.

We identified liver‐derived blood proteins C3, HP, ITIH4, and SAA1 as the candidate molecules associated with cognitive impairment based on the proteomics of mouse liver and blood, as well as the transcriptomic and proteomic profiles of human organs. HP, ITIH4, and SAA1, the major acute‐phase biomarker proteins, may be rapidly upregulated in response to traumatic stimulation or inflammation.^[^ ^22^, ^23^, ^24^, ^41^ ^]^ HP is a hemoglobin‐binding protein, and two classes of alleles at the Hp locus yield 3 genotypes, Hp 1‐1, Hp 2‐1, and Hp 2‐2. The *Hp* polymorphisms were reported to affect cognitive impairment in African American adults with type 2 diabetes,^[^ ^42^ ^]^ and the risk of poor neurological outcomes and cognitive impairment in patients with subarachnoid hemorrhage.^[^ ^43^ ^]^ The rs4687658 in *ITIH4* was found to be related to schizophrenia and intracranial volume.^[^ ^44^ ^]^ Previous study demonstrated that SAA1‐overexpressed mice show the intensified neuronal inflammation and the greater memory decline in APP mice.^[^ ^45^ ^]^ Endogenous C3 protein was elevated in brains of AD patients and models.^[^ ^20^ ^]^ Its overactivation in the brain has been reported to promote AD pathology and cognitive impairment, as well as compromise neuronal morphology and function via C3‐C3aR‐mediated signaling.^[^ ^19^, ^20^, ^21^ ^]^ However, to our knowledge, there have been no reports on whether above peripheral proteins are involved in regulating the CNS and cognitive function.

Our results indicated that anesthesia/surgery can increase serum C3 concentrations in both humans and mice. The changes in serum C3 levels primarily resulted from surgery‐induced release of C3 from the liver, as our experimental evidence showed that anesthesia/surgery only leads to upregulation of *C3* mRNAs in the liver, without affecting the transcription levels of *C3* gene in the brain or other peripheral organs, and the increases in hippocampal C3 levels caused by anesthesia/surgery occurred later than that in the liver and blood. The current evidence strongly confirms that, at the organizational level, the increase of C3 in the hippocampus due to anesthesia/surgery mainly originates from the periphery. This is because the liver‐specific C3 gene knockout mice showed a reduction of ≈50% in the C3 protein levels in their brains after anesthesia/surgery, and we did not observe any changes in the C3 mRNA levels in the hippocampal tissues. However, it is worth mentioning that at the cellular level, we do not rule out the possibility that a small number of hippocampal cells may have increased C3 production due to the effects of anesthesia/surgery, although the amount produced is so small that it is ignored at the tissue level. In the future, it is worth further investigating which hippocampal cells are directly affected by anesthesia and surgery in promoting C3 production. Additionally, it is important to clarify whether changes in C3 levels within hippocampal cells or the C3 that enters the central nervous system from the periphery have a greater impact on POCD. Our previous research determined that anesthesia/surgery can regulate the expression levels of cognition‐related genes by affecting their epigenetic modifications.^[^ ^29^, ^30^ ^]^ In this study, we conducted Bulk‐RNA and targeted bisulfite sequencing to determine that anesthesia/surgery‐induced C3 upregulation in the liver is linked to hypomethylation of CpG sites in *C3* gene promoter.

Generally, the BBB strictly restricts the entry of blood‐derived products into the CNS.^[^ ^31^ ^]^ Therefore, blood‐derived C3 should be unable to directly enter the CNS when the BBB is intact, as its molecular weight reaches 186.40 kDa, unless the BBB is compromised. In the current study, we used TEM to confirm that anesthesia/surgery can disrupt the ultrastructure of the BBB in the aged mice. The biotinylated C3 recombinant protein tracer technology in WT and C3‐KO mice demonstrated that peripheral C3 protein crosses the CNS under the premise of BBB injury. Dynamic tracking of C3 using the iSLET system revealed that the liver‐derived C3 protein can enter the hippocampus through the circulatory system. These findings demonstrate that anesthesia/surgery not only induces BBB breakdown but also results in hepatogenic C3 protein elevations in the blood, and these dual effects of surgical intervention ultimately facilitate the influx of C3 into the brain. BBB disruption is a hallmark of neurodegenerative diseases, containing AD, Parkinson's disease, Amyotrophic lateral sclerosis, Multiple Sclerosis, and Huntington's disease.^[^ ^46^ ^]^ As we known, aging‐ or external factor‐driven systemic inflammation and oxidative stress can lead to the BBB degradation and dysfunction.^[^ ^46^, ^47^ ^]^ Therefore, if BBB has been disrupted by these factors, peripheral C3 proteins could theoretically cross the brain and increase the risk of those neurodegenerative diseases. Effectively controlling the integrity of the BBB could protect cognitive function and slow the progression of the neurodegeneration by blocking harmful peripheral proteins from entering the CNS. In addition, some molecules inhibiting inflammation and oxidative stress may serve as potential drugs to prevent POCD in elderly patients undergoing elective surgery by reduce the BBB degradation.

In this study, we identified that inhibition of liver‐derived C3 can ameliorate cognitive impairment induced by anesthesia/surgery, as well as rescue anesthesia/surgery‐induced neural activity inhibition, impairment of synaptic integrity and plasticity. In addition, we also determined that inhibiting liver‐derived C3 can rescues the cognitive decline of AD. Those findings display that hepatogenous C3 contributes to the impairment of cognitive function. Moreover, we demonstrated that the entry of peripheral C3 into the brain is required for anesthesia/surgery‐induced hippocampal C3‐C3aR signaling activation that promotes abnormal phagocytosis of synapses by microglia. In contrast, the inhibition of liver‐specific C3 can mitigate these processes. Notably, our results demonstrated that inhibiting liver C3 has no significant adverse effects on multiple organs in mice; instead, it can reduce surgery‐induced liver injury. These findings suggest that inhibiting peripheral C3 may have translational potential for cognitive‐related diseases. In the future, studies on peripheral C3 inhibition for the treatment of AD and POCD in primates and clinical settings could be considered to clarify the therapeutic effects and potential risks of C3 inhibition in these clinical diseases. Finally, we observed that perioperative serum C3 elevations caused by anesthesia/surgery or other preoperative factors are related to poor postoperative cognitive function in surgical patients, with a relatively good predictive ability for postoperative POCD. Therefore, peripheral C3 is not only a potential biomarker for predicting cognitive impairment, but also a promising target for the prevention and treatment of cognitive impairment. This also provides a new understanding into the more susceptibility to postoperative POCD in surgical patients with chronic liver diseases than other surgical population.^[^ ^7^, ^8^ ^]^

In summary, our study is the first to demonstrate that protein disruption of the liver mediates cognitive impairment caused by anesthesia/surgery, and future studies should explore the regulatory effects and mechanisms of other hepatogenic blood proteins on cognitive function. Given their impacts on the CNS,^[^ ^9^, ^11^, ^14^, ^16^, ^17^ ^]^ blood proteins could be considered as promising diagnostic and therapeutic targets to increase cognitive resilience as well as salvage cognitive disorders. Our next research will further explore the regulatory mechanisms of epigenetic modifications in those liver proteins. Previous study demonstrated the associations of peripheral changes such as inflammation and immune activation with POCD in elderly people,^[^ ^48^ ^]^ while the details the central mediating mechanisms of these peripheral factors on POCD remain to be extensively investigated. In the future, we need to conduct clinical studies to compare the effect intensity of peripheral C3 and these factors on POCD. Additionally, larger samples of POCD and non‐POCD patients need to be included to further clarify the diagnostic capability of serum C3 in POCD. Our experiment only included male C57BL/6J mice. This choice was made to avoid confounding factors associated with estrous cycle‐dependent immune modulation, align with established models of aging‐related neurodegeneration, and ensure physiological stability during longitudinal procedures. However, this focus limits the generalizability of our findings to female mice and may overlook potential sex‐specific differences in the neurobiological mechanisms underlying our observed outcomes. Although Xie’ team previously confirmed no significant difference in behavioral performance and certain molecular levels (e.g., NR2B and IL‐6) in the brain and blood between male and female mice with anesthesia/surgery,^[^ ^49^, ^50^ ^]^ future studies should incorporate female mouse models to provide a more comprehensive understanding of C3 dynamics and their implications across sexes. Moreover, the associations of peripheral HP, ITIH4, and SAA1 proteins with cognitive function could be further investigated.

In conclusion, this study confirms that the liver mediates the anesthesia/surgery‐induced cognitive impairment. Our findings provide compelling evidence that anesthesia/surgery can induce the BBB breakdown and enhance the release of liver‐derived C3 protein into the blood. This surgical dual factors ultimately causes C3 to smoothly cross the damaged BBB to selectively colocalize with C3aR in the hippocampus. Inhibition of hepatogenic C3 rescues anesthesia/surgery‐induced cognitive impairment, structural and functional injury of synapse, and C3aR‐mediated microglial phagocytosis, highlighting peripheral C3 as a promising target for the prevention and therapy of cognitive impairment. Changes in serum C3 during the perioperative period are associated with postoperative cognitive impairment in surgical patients, with potential for predicting POCD.

## 4 Experimental Section

The study design and experimental flowchart is presented in Figure S14 (Supporting Information).

### Surgical Participants and Evaluation of POCD‐Ethics and Inclusion Criteria

This study was approved in April 2023 by Shanghai Fourth People's Hospital Affiliated to Tongji University School of Medicine Ethics Board (reference number 2023043‐001), and has been registered in the Chinese Clinical Trial Registry (Registration number: ChiCTR2300073351). Each participant received a written informed consent. Elective elderly surgical patients who would undergo open abdominal/thoracic or laparo‐/thoracoscopic surgery with general anesthesia from the year of 2023 to 2024 were included in this study. All patients received combined intravenous‐inhalational anesthesia, induced with propofol, sufentanil, and rocuronium for muscle relaxation, and maintained with continuous propofol infusion and/or sevoflurane. Postoperative analgesia was provided with patient‐controlled intravenous analgesia (PCIA). Both groups of patients did not receive drugs such as dexmedetomidine during the perioperative period that could potentially affect postoperative cognition. All included participants had no preoperative cognitive impairment, which was identified by the Mini‐Mental State Examination (MMSE) assessment according to the diagnostic criteria for cognitive impairment (MMSE score < 27).^[^ ^51^ ^]^ In addition, clinical features of participants were collected, containing age, sex, education, operation time, body mass index, and medical history. Participants have no any of the following diseases, including neurological diseases, familial hereditary diseases, liver or kidney dysfunction, metabolic diseases, other cancers other than stomach and lung cancer. The detail clinical information of surgical patients was shown in Table S10 (Supporting Information).

### Assessment of POCD

All included participants were not diagnosed with postoperative delirium according to the diagnostic criteria for delirium,^[^ ^52^ ^]^ and received the Modified Telephone Interview for Cognitive Status (TICS‐M) conducted by our professional cognitive function evaluator 1 year after surgery. The TICS‐M is one of the most frequently applied and well‐established telephone cognitive assessment tools for POCD diagnosis,^[^ ^53^, ^54^ ^]^ with an established cut‐off of < 32 out of 50 for classifying cases of cognitive impairment.^[^ ^55^ ^]^ According to well‐established diagnostic criteria for POCD using TICS‐M,^[^ ^53^, ^54^, ^55^ ^]^ participants with TICS‐M < 32 were diagnosed with POCD, while those who with TICS‐M ≥ 32 are classified as non‐POCD.

### Collection of Human Blood

Fasting morning blood (2–5 mL) of each participant was collected 1 day before and 1 day after surgery and placed into a vacuum collection vessel, leaving for 30–60 mins at 20 °C. Serum was obtained through centrifuging the supernatant of the collection vessel at 3000 r min^−1^ for 5 min, and stored at −80 °C.

### Mice

C57BL/6J male mice aged 18 months were purchased from Shanghai Model Organisms Center, Inc. Six‐month‐old 5×FAD male mice were purchased from Cyagen Biosciences(C001541). C3‐KO mice aged 3 months were obtained from Cyagen Biosciences (S‐KO‐01270). Additionally, *Albumin‐creER^T2^; C3^loxp/loxp^* double‐transgenic (cKO) mice (hepatic‐C3 conditional knockout mice) were generated by crossing *Albmin‐CreER^T2^* mice (C001488) with *C3^loxp/loxp^* mice (S‐CKO‐01478) that are purchased from Cyagen, Shenzhen, China. To excise the loxp sites using Cre recombinase, 2‐month‐old male mice were intraperitoneally injected with 100 mg/kg tamoxifen (Sigma T5648) for 7 consecutive days (once daily).

All mice were housed under standard environmental conditions (a 12‐h light/dark cycle, a temperature of 24 ± 1 °C, and 55 ± 5% relative humidity) with free access to food and water. All experiments were conducted in accordance with the Chinese Council on Animal Care Guidelines and approved by the Tongji University Animal Ethics Committee (TJBH00921101).

### Virus Vector Construction and Injection

Recombinant adeno‐associated virus (rAAV‐TBG‐mCherry‐shRNA(C3)‐WPREs) and control scramble virus (rAAV2/8‐TBG‐mCherry‐shRNA (Scramble)‐WPREs) were designed by BrainVTA Technology (Wuhan, China). The detailed principle of specific intervention gene expression in the liver was summarized in the previous report.^[^ ^17^ ^]^ A total of 200 µL of rAAV2/8 viral solution (1 × 10^12^ genomic copies/mouse) was injected to each mouse via the tail vein for 3 weeks, referring to previous experimental procedures.^[^ ^17^ ^]^

### Biotinylated C3 Injection

Biotinylated C3 (20 ng) diluted in saline solution (MCE) was administered through tail vein injection in mice 24 h after anesthesia/surgery and allowed to circulate for 6 h. The mice were then anesthetized with 2% isoflurane (Baxter Healthcare, Puerto Rico, USA) and perfused with   PBS. Their brains were fixed overnight in 4% PFA, cryoprotected with 30% sucrose and frozen, and then sliced at a thickness of 40 µm. C3‐biotin conjugate was detected with Oregon Green‐488 conjugate of NeutrAvidin biotin‐binding protein (Thermo Fisher Scientific). After three washes with PBS, slices were stained with DAPI, mounted, and imaged using an Olympus FV3000 confocal microscope and FV31S‐SW software.

### Surgical Models

Laparotomy and internal fixation surgery of tibial fractures, two classic surgical methods, were used to build POCD model.^[^ ^26^, ^27^ ^]^ Eighteen‐month‐old mice (25–30g) underwent laparotomy or internal fixation surgery of tibial fractures under isoflurane anesthesia.

### Laparotomy

Mice were first anesthetized with 2% isoflurane (Baxter Healthcare, Puerto Rico, USA) in oxygen at 1 L min^−1^ for induction for 2 min, and then continued to be anesthetized with 1.4%–2% isoflurane in oxygen at a rate of 1 L min^−1^ after the reversal reflex disappeared, as previously described.^[^ ^26^, ^56^ ^]^ Mice were then placed on a heated surgical table to maintain the core body temperature at 37.5 °C. The subsequent operations referred to our previous experimental procedures.^[^ ^26^, ^56^ ^]^ Briefly, a ≈1‐cm abdominal incision was made from the xiphoid to the proximal pubic symphysis after shaving abdominal hair and disinfection. A 5‐cm segment of the small intestine was gently pulled out from the surgical cavity and wrapped in gauze moistened with sterile saline. Subsequently, the small intestine was gently kneaded for 10 mins to mimic clinical exploratory laparotomy. After surgery, the muscle and skin were closed layer by layer using 4/0 absorbable sutures (VICRYL; Ethicon, USA). Topical EMLA cream (2.5% lidocaine and 2.5% prilocaine) was used at the end of the procedure. The surgical procedures were performed under sterile conditions and lasted ≈30 mins.

### Internal Fixation Surgery of Tibial Fractures

The anesthesia method is the same as the above abdominal surgery. The surgical method was shown as previously described.^[^ ^27^ ^]^ Briefly, A 0.4–0.6 cm vertical incision was made near the tibial tubercle in mice, and a needle was inserted into the tibial tubercle. After the surgery, buprenorphine was administered subcutaneously to alleviate pain.

### Behavioural Testing‐Open Field

This test was performed to detect spontaneous locomotor activity and anxiety‐like behaviour of mice.^[^ ^56^ ^]^ On the first day after surgery, mice were brought to the testing room 30 mins before the experiment to familiarize themselves with the environment. Mice were gently placed on the center in a rectangular chamber (40 × 40 × 40 cm) composed of gray polyvinyl chloride, and were allowed to freely explore for 5 mins. The average speed and the time spent in the central area were quantified blindly using Smart Video Tracking Software (Panlab; Harvard Apparatus) to assess motor ability and anxiety, respectively.

### Barnes Maze Test

This test was carried out as described in our previous work.^[^ ^26^, ^56^ ^]^ Briefly, the escape compartment was removed after 4 consecutive days of the training phase (3 trials per mouse on day 1–4 after surgery), and the testing phase was conducted for 2 mins on day 5 after surgery. All sessions were videotaped and analyzed blindly. The time required to enter the escape compartment as well as the percentage of time spent in the target quadrant, were recorded to evaluate the learning and memory ability of mice. The chamber was cleaned before/after each session with 75% ethanol. All data were processed using Any‐Maze (Stoelting, San Diego, USA).

### Fear Conditioning Test

This test was used to examine contextual fear memory of mice, as this previous description.^[^ ^56^ ^]^ On the day of habituation, the mice were allowed to move freely in the conditioning chamber for 10 mins. On the day of training (day 6 after surgery), the 2 min period prior to the first shock was utilized to assess baseline freezing, and mice suffered five‐foot shocks (0.8 mA for 2 s; interval, 35–60 s). Then, all mice were returned to their home cages. Twenty‐four hours later, those mice were put into the same chamber for 5 mins to assess contextual memory retrieval. Freezing was defined as no movement for 2 s. To eliminate odor cues, the apparatus was carefully cleaned with 75% ethanol between each test. The freezing time was recorded using ANY‐maze Software (Stoelting Co.).

### Morris water Maze (MWM) Test

This test was conducted to assess spatial learning and memory. All mice underwent training for a duration of 5 days, during which they swam four times each day to locate a hidden platform. Each mouse was given 90s to find the concealed platform and was allowed to remain on it for 20s before being removed. If a mouse was unable to locate the platform within the allotted time, it was guided to the platform and given an additional 20s to rest. We recorded the time taken by each mouse to reach the platform, referred to as escape latency, and used this measurement to evaluate learning and memory. On the sixth day, the mice were placed in the third quadrant, and video software recorded their search trials for 90s to track their movements toward the platform's location. We assessed each mouse's memory by measuring the time spent in the target quadrant, as well as the total number of crossings into the platform area. Subsequently, we compared the measurements of learning and memory, as well as swimming speeds, among all groups of mice. The swimming processes were recorded, and the trajectory was analyzed using Ethovision XT.

### Collection of Mouse Blood

Blood of mice were collected by eyeball bleeding after deep anesthesia. Serum was obtained from the blood, and the procedures of serum collection were the same as that of surgical patients. Plasma was obtained from the blood according to the following procedures: The blood was quickly transferred into a heparinized centrifuge tube and let it stand at room temperature (25 °C) for 1h. Then, the blood was centrifuged for 15 mins at 1000 × g at 4 °C. Next, the supernatant was carefully transferred into a clean 1.5 mL centrifuge tube and centrifuged at 16000 × g for 10 mins at 4 °C. The supernatant was gently aspirated into a new cryopreservation tube and stored at −80 °C.

### Mouse Organ Acquisition

Mice were anesthetized and perfused with 0.9% saline. The fresh hippocampus, liver, heart, kidneys, spleen, and lungs were acquired and used for qPCR, Western blot, and ELISA.

### 4D Label‐Free Proteomics‐LC‐MS/MS

4D Label‐Free Proteomics was performed in the paired‐sample liver tissues and plasma from control and surgery mice at 1 day after laparotomy surgery by H‐Wayen Co., Ltd (Shanghai, China). The experimental procedures referred to previous studies.^[^ ^57^, ^58^ ^]^ Briefly, the minced liver tissues were quickly lysed in lysis buffer. The liver lysate and plasma were centrifuged at 14000×g for 10 mins to collect the supernatant. Protein concentrations was measured with bicinchoninic acid assay. Extracted proteins were digested after precipitation of proteins. The precipitation was centrifuged at 4 °C at 8000×g for 10 mins for collecting the precipitate. According to the amount of protein, we add the corresponding volume of enzymolysis diluent to redissolve the protein precipitate. The fractions were finally analyzed using liquid chromatography tandem mass spectrometry (LC‐MS/MS). Abundance of proteins was normalized to obtain the quantitative values, which was used for the subsequent analyses.

### Differential Protein Analysis

A log2 transformation was performed on the normalized quantitative matrix. Differential protein analysis was conducted for the log2‐converted quantitative values among two groups of mice with the limma package v3.50.3. The |log (fold change)| > 0.25 and Benjamini‐Hochberg adjusted *P* value < 0.05 were applied as the cutoff for screening differential proteins.

### Weighted Gene Co‐expression Network Analysis (WGCNA)

WGCNA can be used for finding modules (clusters) of highly associated proteins using the cluster eigengene or the intramodular hub molecules and for calculating the relationships of module membership (molecule) with phenotypes.^[^ ^59^ ^]^ This tool was utilized to determine the critical liver and plasma proteins associated with surgery mice according to this analysis process.^[^ ^59^ ^]^ The normalized quantitative matrixes were used to construct a protein co‐expression network and further identify the highly related protein modules closely correlated with surgery mice.

### Origin Identification of Liver‐derived Blood Proteins

To identify the main organic origins of the above proteins associated with surgery mice, we evaluated their mRNA and protein profiles of human organs. First, we assessed liver‐specificity origins of those proteins based on tissue specificity scores of 126 liver‐derived blood proteins that show a tissue‐restricted expression in the liver and are predicted to translocate to peripheral blood and have systemic effects on the human body,^[^ ^9^, ^60^, ^61^ ^]^ which was downloaded from Human Protein Atlas (HPA) database (https://www.proteinatlas.org/). Furthermore, gene TPM expression data (GTEx_Analysis_2017‐06‐05_v8_RNASeQCv1.1.9_gene_tpm.gct) from 11 584 male and 5878 female samples consisting of brain 13 regions and 30 peripheral organs was obtained from GTEx database (https://www.gtexportal.org/home). A log2 transformation was performed on mRNA expression matrix and calculated mean value of code gene for each protein. The gganatogram package v. 1.1.1 and pheatmap package v. 1.0.12 were applied to display transcription profiles of genes.

### Single‐Cell RNA Sequencing (scRNA‐seq) of the Hippocampus‐Mouse Hippocampus

Mice injected with either rAAV‐Scramble or rAAV‐shRNAC3 (3 mice per group) were lethally anesthetized using isoflurane and subsequently perfused with cold PBS to eliminate any unwanted blood cells from the target tissues. Hippocampi of those mice were clipped and stripped of impurities or dead tissues. The generation of single‐cell suspensions and cell capture was conducted following previously established protocols.^[^ ^62^ ^]^

### Library Construction and High‐Throughput Sequencing

The freshly prepared single‐cell suspension was adjusted to a concentration of 700–1200 cells µl^−1^. Library preparation and loading were carried out according to the manufacturer's protocol for the 10× Genomics Chromium Next GEM Single Cell 3ʹ Reagent Kits v3.1 (cat. no. PN‐1000268). The constructed libraries were then sequenced on the Illumina NovaSeq 6000 PE150 platform for high‐throughput sequencing. The sequencing was provided by OE Biotech Co., Ltd. (Shanghai, China).

### scRNA‐seq Data Preprocessing, Quality Control, and Cell Annotation

The FASTQ files were processed and aligned to the CRCm39 mouse reference genome using Cell Ranger software version 9.0.0 from 10x Genomics, summarizing unique molecular identifier (UMI) counts for each barcode. The resulting UMI count matrix was then analyzed with the R package Seurat version 4.0.0. To eliminate low‐quality cells as well as potential multiplet captures, several filtering criteria were applied: Cells were filtered based on the number of genes (genes < 200), log10 of genes per UMI (log10GenesPerUMI < 0.7), UMI counts (UMIs < 1000), the percentage of hemoglobin RNA UMIs (proportion of hemoglobin genes > 5%), and the percentage of mitochondrial RNA UMIs (proportion of mitochondrial genes > 10%). Moreover, the DoubletFinder package version 2.0.3 was utilized to remove potential doublets. To obtain normalized gene expression data, library size normalization was performed using the NormalizeData function. The global‐scaling normalization method “LogNormalize” was used to normalize the gene expression measurements for each cell by the total expression (a scaling factor is 10000 by default), as well as log‐transform the results.

We identified the top 2000 highly variable feature genes in each sample using the Seurat function “FindVariableFeatures” with the vst method. Principal Component Analysis (PCA) was conducted to reduce dimensionality using the RunPCA function. Next, batch effects were addressed by applying the RunHarmony function from the R package Harmony version 1.0. Graph‐based clustering was performed to categorize cells based on their gene expression profiles using the FindClusters function (resolution = 0.5). Cell visualization was achieved through a 2D Uniform Manifold Approximation and Projection (UMAP) algorithm with the RunUMAP function. The FindAllMarkers function (test.use = wilcox) was employed to identify marker genes for each cluster. Cell types were determined using a combination of marker genes sourced from the literature, the Mouse Brain Atlas (http://mousebrain.org/), the Mouse RNA Atlas (https://bis.zju.edu.cn/MCA/), and CellMarker 2.0 (http://117.50.127.228/CellMarker/index.html).

### Differential Expression Analysis

Differentially expressed genes (DEGs) were identified using the FindMarkers function (test.use = MAST). The Benjamini‐Hochberg adjusted *P* value < 0.05 and |log2 fold change| > 0.25 was established for determining significant differential expression.

### Gene Enrichment Analysis and Cognition‐Related Biological Function Analysis

Gene Ontology (GO) enrichment and KEGG pathway enrichment analyses of upregulated and downregulated DEGs were conducted using R packages clusterProfiler version 4.2.2 and org.Mm.eg.db. We focused on the well‐known cognition‐related biological functions including interleukin 1‐mediated signaling pathway, response to interleukin‐1, phagocytosis, phagocytosis engulfment, I‐kappaB kinase/NF‐kappaB signaling, toll‐like receptor signaling pathway, NIK/NF‐kappaB signaling, synaptic vesicle cycle, synapse organization, cell chemotaxis, activation of immune response, Wnt signaling pathway, neuron death, learning, learning or memory, and response to oxidative stress. We constructed a matrix.gmt of above biological functions and DEGs among those functions referencing to instruction of scMetabolism v2.1.0 package.^[^ ^63^ ^]^ The AUCell activity scores of those biological functions based on single‐cell expression matrices were calculated using scMetabolism. In addition, the activity of single‐cell biological functions was estimated based on AUCell scores of those biological functions, as the previously described.^[^ ^63^ ^]^ Differentially biological functions were determined with FindAllMarkers function (MAST test) based on single‐cell AUCell scores within each cell type. Bonferroni‐adjusted *P* value < 0.05 and |log_2_FC| > 0.25 was regarded as the cutoff.

### Bulk‐RNA SEQUENCING

Bulk‐RNA sequencing was conducted in the liver (control and surgery mice) and hippocampus tissues (surgery mice injected with rAAV‐Scramble or rAAV‐shRNAC3) on the first day after surgery. Total RNA was extracted with the TRIzol reagent (Invitrogen, CA, USA). RNA purity, quantification, and integrity were measured. Then the libraries were constructed using RNA‐seq Library Prep Kit. The libraries were finally sequenced on an Illumina Novaseq 6000 platform. The transcriptome sequencing was carried out by OE Biotech Co., Ltd. (Shanghai, China). Differential gene analysis was performed to identify the differential expressed genes (DEGs) between two groups of mice using the edgeR package. The |log fold change| > 0.58 and Bonferroni‐adjusted < 0.05 were applied as the cutoff for screening DEGs.

### Targeted Bisulfite SEQUENCING

The target bisulfite sequencing in *C3* gene was carried out in the liver tissues from the same batch of mice used for Bulk‐RNA sequencing. This sequencing was conducted by E‐Gene Co., Ltd (Shanghai, China). Specific promotor regions were selected and bisulfite sequencing PCR primers were designed through online MethPrimer software (www.urogene.org/methprimer/index.html). These primers were designed to identify regions without CpG sites to avoid amplification bias between methylated and unmethylated sequences (Table S11, Supporting Information). The subsequent bisulfite conversion of genomic DNA, two rounds of PCR for targeting promotor regions, as well as sample barcoding, were described in our previous study.^[^ ^29^ ^]^ Briefly, the liver tissues were incubated with DNA lysis buffer, and DNA was extracted with phenol‐chloroform method. Then, genomic DNA was converted with ZYMO EZ DNA Methylation‐Gold Kit™ (Zymo) based on the manufacturer's instructions. Furthermore, bisulfite‐converted DNA was amplified utilizing EpiTaq HS DNA polymerase (Takara) with primers targeting *C3*, followed by a second round of amplification using Phusion DNA polymerase. The amplificated PCR products were further applied for library construction. Finally, those barcoded libraries were quantified by Agilent 2100 Bioanalyzer and sequenced by Illumina Nova‐seq 6000 instrument. The differentially methylated CpG sites were identified by comparing their degree of methylation in *C3* gene promoter with t‐test and R package limma v3.50.3. The Bonferroni‐adjusted < 0.05 was considered as the cutoff.

### Enzyme Linked Immunosorbent Assay (ELISA)

According to the manufacturer's protocols, the levels of C3 protein (Mouse Complement C3 Kit, JL10274; Human Complement C3 Kit, JL10735) were measured. The concentrations of C3 per sample were obtained from the average of the three tests for each sample.

### H&E Staining of the Heart, Liver, Spleen, Lung, and kidney in Mice

The heart, liver, spleen, lung, and kidney were harvested and fixed in 10% buffered formalin solution for paraffin embedding. Hematoxylin and eosin (H&E) staining of 3 µm thickness liver sections were carried out using standard protocols. The fibrosis area and the total area investigated were evaluated with Image J software from the image fields of each slide.

### Western Blotting

The harvested tissues were infused using 1 x PBS to wash out the contents. Subsequent experimental steps were described in our previous work.^[^ ^30^ ^]^ In brief, tissues were lysed to product lysates, and lysates were centrifuged at 12000 × g for 15 min at 4 °C. Total protein concentration was quantified with Pierce protein assay kit (Cat# 23 225, Thermo Fisher). Proteins were separated on 10% SDS‐PAGE gels and transferred onto a fit PVDF membrane (Merck Millipore). The membranes were blocked by 5% (v/v) skimmed milk and incubated at 4 °C overnight with anti‐C3 (1:2000, AB200999, Abcam), anti‐C3aR (1:1000, AB126250, Abcam), anti‐β‐actin (1:3000, 20536‐1‐AP, Proteintech), anti‐PSD95 (1:1000, 30255‐1‐AP, Proteintech), anti‐synaptophysin (1:1000, 67864‐1‐Ig, Proteintech), streptavidin‐HRP (1:15 000, 21 126, Thermo), ZO‐1 (1:5000, 21773‐1‐AP, Proteintech), Occludin (1:5000, 27260‐1‐AP, Proteintech), C5aR (1:2000, 21316‐1‐AP, Proteintech), CD11b (1:2000, 31745‐1‐AP Proteintech). After incubation with HRP‐conjugated secondary antibodies (1:3000, AB_2 722 564, Proteintech), all samples were normalized to β‐actin. Finally, all quantifications were visualized with the ChemiDoc XRS+ system (Bio‐Rad).

### qPCR

Total RNA was extracted using TRIzol regent (R411‐01, Vazyme) according to the manufacturer's instructions, and RNA purity and concentration were assessed by Nanodrop. Next, mRNA was reverse‐transcribed into cDNA using the qRT SuperMix reverse transcription kit (R333, Vazyme). Then, qPCR was performed using SYBR Green Master Mix (Q712‐02, Vazyme) on a Quant Studio 1 Real Time PCR system (Thermo Fisher, USA). Gapdh was used as the control. The following primers were used: *C3*, Forward 5′‐CCAGCTCCCCATTAGCTCTG‐3′ and Reverse 5′‐GCACTTGCCTCTTTAGGAAGTC‐3′. Gapdh, Forward 5′‐AGGTCGGTGTGAACGGATTTG‐3′ and Reverse 5′‐TGTAGACCATGTAGTTGAGGTCA‐3′. The three Ct values were averaged, and the data were displayed as the geometric mean of the housekeeping gene (GAPDH) using the Ct delta method (2‐ΔΔCt).

### Immunofluorescence Staining

Immunofluorescence staining was performed referencing to our previous experimental process.^[^ ^64^ ^]^ Briefly, the brain or liver tissues were fixed with 4% paraformaldehyde (PFA) following perfusion for 24h. Tissues were subsequently immersed in 30% sucrose solution for 3 days and cut into 40 µm sections by freezing microtome. These sections were then washed in 0.1 M PBS 3X for 10 mins. Sections were then blocked in 5% donkey serum containing 0.5% Triton X‐100 for 1h at room temperature, and then incubated for overnight at 4 °C with the following primary antibody: rabbit anti‐C3 (1:2000, AB200999, Abcam), rabbit anti‐C3aR (1:1000, AB126250, Abcam), rabbit anti‐Iba1 (1:1000, 019–19741, FUJIFILM Wako), Rat anti‐CD68 (1:500, MCA1957, Bio‐Rad), rabbit anti‐PSD95 (1:1000, 30255‐1‐AP, Proteintech), and mouse anti‐synaptophysin (1:1000, 67864‐1‐Ig, Proteintech), mouse anti‐NeuN (1:500, MAB377, Milipore), Chicken anti‐GFAP (1:500, ab4674, Abcam), Anti‐V5 (1:500, R960‐25, Invitrogen). Furthermore, sections were incubated with DAPI and the corresponding secondary antibodies to bind to the primary antibodies: goat anti‐rabbit Alexa Fluor 488 (1:500, ab150077, Abcam), goat anti‐rabbit Alexa Fluor 594 (1:500, ab150080, Abcam), goat anti‐mouse Alexa Fluor 488 (1:500, ab150113, Abcam), and goat anti‐rat Alexa Fluor 488(1:500, ab150157, Abcam), Streptavidin‐Alexa Fluor 647 IgG (1:500, S21374, Invitrogen). Ultimately, sections were mounted and imaged using an Olympus FV3000 confocal microscope and FV31S‐SW software.

### Dynamic Tracking of Liver‐Derived C3

To label liver‐derived secretory proteins, we adopted the iSLET (in situ Secretory protein Labeling via ER‐anchored TurboID) system, a previously reported and validated method that enables in vivo labeling of secretory proteins via ER‐anchored proximity biotinylation.^[^ ^33^ ^]^ The Sec61b‐V5‐TurboID construct (Addgene, #166 971) was used to generate AAV vectors, which were synthesized by BrainCase (China). ≈10^8^ adenoviral GFP or Sec61b‐TurboID particles were injected into mice via the tail vein. 24 mg mL^−1^ biotin stock was prepared in DMSO. Vehicle (10% DMSO in PBS) or Biotin solution (2.4 mg mL^−1^) was filtered through a 0.22 µm PES syringe filter and injected 10 µL g^−1^ (24 mg kg^−1^) by daily intraperitoneal injection for 3 consecutive days. Biotin was not administered on the last day to minimize residual biotin in the blood. Blood, hippocampus, and liver samples were obtained after deep anesthesia.

Immunoprecipitation (IP) was used to detect streptavidin‐labeled C3 protein in plasma and tissue samples. Protein samples were extracted using lysis buffer with protease and phosphatase inhibitors, and supernatants were collected by centrifugation at 14000 rpm for 15 min at 4 °C. The lysates were incubated overnight at 4 °C with either anti‐C3 antibody or streptavidin‐conjugated magnetic beads. The next day, protein A/G Sepharose beads were added for 2 h at 4 °C if necessary. After washing with ice‐cold buffer, proteins were eluted by boiling in loading buffer and analyzed by Western blotting.

### Stereotactic Intracerebral Injection

As previously described,^[^ ^30^ ^]^ mice were first anesthetized by 2% isoflurane, and rAAV‐hSyn‐GCaMp6s (400nl, BrainVTA, China), rAAV‐CAG‐DIO‐mCherry, and scramble virus were then bilaterally injected into the dorsal hippocampus (AP: −1.50 mm; ML: ±1.70 mm; DV: −1.75 mm) at a speed of 100 nL min^−1^. The syringe was finally left for an additional 10 mins in place before the incision was sutured.

### In Vivo Fiber Photometry Recording

Calcium signals in the hippocampus were collected in the commercialized fiber photometry system (RWD, China). At 21 days before recording, an optic fiber coated with a ceramic ferrule (diameter: 1.25mm, RWD, China) was implanted into the hippocampus (AP: −1.50 mm; ML: ± 1.70 mm; DV: −1.70 mm). During imaging, a 470nm LED (40mW at fiber tip) was used for excitation, while calcium in dependent signals were obtained using a 410nm LED (20mW at fiber tip) to correct for movement artifacts. The fluorescence signals were normalized to calculate the fluorescent change (△F/F0), where F0 was the baseline fluorescent level. During behavioral recording, each event trace was extracted with the reference to the tag (from −2 s to + 10 s relative to the freezing time in the fear conditioning test). Relative fluorescence was converted to the Z‐score and presented as the heatmap series.

### Electrophysiological Recording

The experimental procedure was based on our previous study.^[^ ^30^ ^]^ Hippocampal slices (400 µm) were prepared from 18‐month‐old mice by a vibratome slicer (VT1200S, Leica) and incubated in artificial cerebrospinal fluid (ACSF) for at least 1 h before use. Slices were transferred to the recording chamber and visualized via infrared‐differential interference contrast microscopy. A concentric bipolar electrode (FHC, Lot#300 125) was positioned in the stratum radiatum of CA1 to stimulate the afferent Schaffer collateral‐commissural pathway from the CA3‐CA1 regions. Then, fEPSPs were recorded in the CA1 region using micropipettes, and LTP was induced by delivery of HFS (100 Hz, 20‐s interval, four trains). Data were obtained with a MultiClamp 700B (Axon Instruments) and analyzed with Clampex 10.7.

### Transmission Electron Microscopy (TEM)

Before sampling, petri dishes with fixative for TEM should be prepared in advance, small tissue blocks were removed from the animal body and immediately put into petri dishes, and then cut into small size of 1 mm^3^ in the fixative. The 1mm^3^ tissue blocks were transferred into an EP tube with fresh TEM fixative for further fixation, which was fixed at 4 °C for preservation and transportation. Then tissues were washed using 0.1 M PB (pH 7.4) for 3 times, 15 mins each. Tissues avoid light post fixed with 1% OsO4 in 0.1M PB (pH 7.4) for 2h at room temperature. After removing OsO4, the tissues are rinsed in 0.1M PB (pH 7.4) for 3 times, 15 mins each. Then, tissues were dehydrated at room temperature and embedded in resin. The resin blocks were sectioned into 1.5µm slices using a semi‐thin microtome, subsequently stained with toluidine blue, and positioned under a light microscope for positioning. Ultimately, these sections were used for ultrathin section and staining. The BBB were observed on a transmission TEM.

### Blood Routine Analyses and Liver Function Injury

The blood routine was measured with Sysmex XN (SYSMEX Co., Ltd., Japan). Liver function injury was assessed by detecting alanine aminotransferase (ALT), aspartate aminotransferase (AST), total bilirubin (TBIL), and direct bilirubin (DBIL) in the serum of mice. Renal function was assessed in mice following anesthesia/surgery by measuring blood urea nitrogen (BUN), serum creatinine (CREA), and uric acid (UA) levels.

### C3aR Antagonist Treatment

C3aR antagonist SB290157 (C3aRA) was purchased from Calbiochem (catalog No. SB290157). The 18‐month‐old C57 mice were intraperitoneally (i.p.) injected with C3aRA (1 mg/kg) or 0.5% dimethyl sulfoxide (vehicle) 1 h pre‐anesthesia/surgery, and 2 and 4d post‐anesthesia/surgery.

### POCD Prediction

Based on serum C3 levels at 1 day after surgery and the delta value of postoperative and preoperative changes in C3, receiver operating characteristic (ROC) curve analysis was used for POCD prediction with R package pROC v1.18.0.

### Statistical Analysis

R v4.3.0 was used for statistical analysis. R package ggplot2 v3.4.4 was used for drawing plots. Two‐tailed Student's t test (normal distribution) or Wilcoxon test (non‐normal distribution) was used for comparisons between the two groups. One‐way ANOVA &Tukey HSD post hoc test (normal distribution) or Kruskal‐Wallis Test & Dunn's test (non‐normal distribution) was used for multiple comparisons between the 3 or 4 groups. Two‐way repeated‐measures ANOVA with Bonferroni's post hoc test for time × group comparisons. Spearman analysis was performed on two groups of continuity variables. All data are represented as mean ± SEM. *P* p‐value < 0.05 was considered statistically significant.

## Conflict of Interest

The authors declare no conflict of interest.

## Author Contributions

Q.W., P.C., and Z.L. contributed equally to this work and shared the first author. X.H., D.W., and L.X conceived and led this study. Q.W., P.C., and Z.L. performed the biochemistry experiments. Q.W. and P.C performed the Bulk‐RNA sequencing experiment and 4D Label‐Free Proteomics. Q.W., P.C., Y.Z., H.W., Q.Z., H.Z., and Z.L. performed animal studies. Q.M., and Z.C. performed histological analysis. P.C., L.T., and Y.L collected clinical samples. E.F., Q.C., X.H., and Y.Z. performed computation analysis. Q.W., P.C., and Z.L. collected and analyzed the data. X.H. wrote the draft. X.H., D.W., and L.X discussed and revised the manuscript. All authors read and approved the manuscript.

## Ethics Approval Statement and Consent to Participate

The animal experiments in this study were approved by the Ethics Committee of Tongji University (Approval Number: TJBH00921101). Cognitive function assessment, blood sample collection, and protein detection conducted in surgical patients were approved by Shanghai Fourth People's Hospital Affiliated to Tongji University School of Medicine Ethics Board (Approval Number: 2023043‐001), and have been registered in the Chinese Clinical Trial Registry (Registration number: ChiCTR2300073351). Each participant received a written informed consent.

## Supporting information



Supporting Information

Supplemental Table

## Data Availability

The authors declare that all data supporting the results of our study are available within the paper and its supplementary information files. Additional datasets supporting the findings of this study are available from the corresponding author upon reasonable request. Transcriptome and protein‐related datasets for human organs can be obtained separately from GTEx database (https://www.gtexportal.org/home) and Human Protein Atlas (HPA) database (https://www.proteinatlas.org/).
