# Supplementary material for: Inhibiting Liver‐Derived C3 Protein Rescues Anesthesia/Surgery‐Induced Cognitive Impairment, Synaptic Disorders, and Microglial Phagocytosis
Source: Adv Sci (Weinh). 2025 Nov 12;13(2):e02034. doi: 10.1002/advs.202502034 (PMC12786287; doi:10.1002/advs.202502034)
Supplement: Supplementary file 1 — Supporting Information [file ADVS-13-e02034-s002.docx]

Supporting Information

**Inhibiting** **Liver-derived Blood C3 Protein** **Rescues Anesthesia/surgery-induced** **Cognitive Impairment, Synaptic Disorders, and Microglial Phagocytosis**

***Authors:*** *Qianqian Wu^1,#^, Peilin Cong^1,#^, Zhouxiang Li^1,#^, Yuxin Zhang^1^, Huanghui Wu^1^, Qian Zhang^1^, Yawei Li^2^, Li Tian^1^,* *Qingyuan Miao^1^, Yinggang Zheng^1^,* *Hui Zhang^1^, Qian Chen^1^, Enduo Feng^1^, Xinyang Li^1^, Zheping Chen^1^, Dong-Xin Wang^2,*^, Xinwei Huang^1,*^, Lize Xiong^1,*^*

***Affiliations:***

^1^Shanghai Key Laboratory of Anesthesiology and Brain Functional Modulation, Clinical Research Center for Anesthesiology and Perioperative Medicine, Translational Research Institute of Brain and Brain-Like Intelligence, Department of Anesthesiology and Perioperative Medicine, Shanghai Fourth People's Hospital, School of Medicine, Tongji University, Shanghai 200434, China

^2^Department of Anesthesiology, Peking University First Hospital, Beijing 100034, China.

^#^Qianqian Wu, Peilin Cong, and Zhouxiang Li contributed equally to this work and shared the first author.

*Corresponding authors: Dong-Xin Wang, dxwang65@bjmu.edu.cn; Xinwei Huang, [huanggenetics@tongji.edu.cn](mailto:huanggenetics@tongji.edu.cn) or huanggenetics@163.com; Lize Xiong, lizexiong@tongji.edu.cn or [mzkxlz@126.com](mailto:mzkxlz@126.com).

**Supplementary figure**


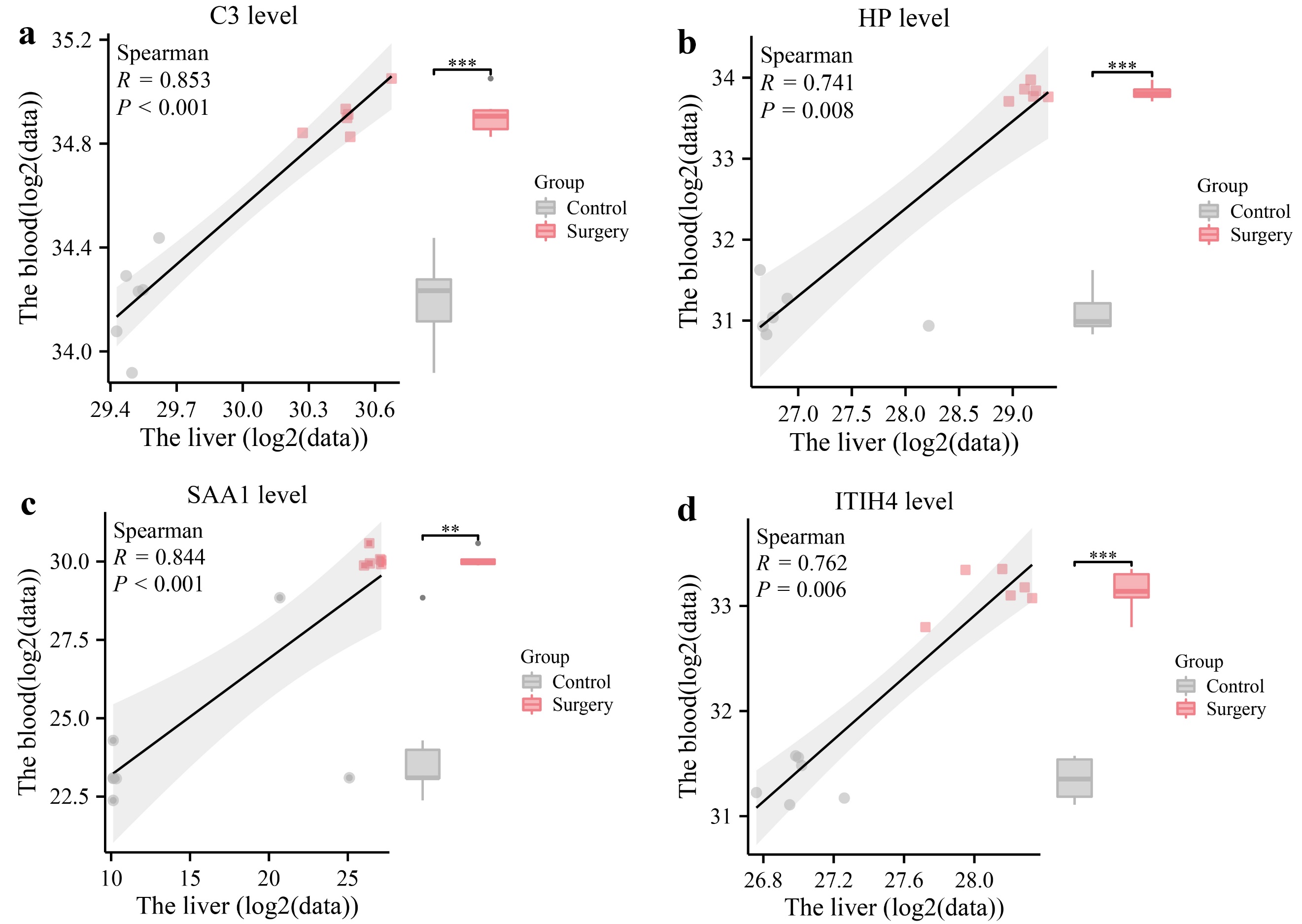


**Figure S1 Spearman correlation analysis for C3, HP, SAA1, and ITIH4 protein levels between the liver and blood.** (a-d) The correlation for each protein levels between the liver and blood. Differential protein analysis was conducted by limma and proteins with |log (fold change)| > 0.25 and *P* < 0.05 were regarded as differential proteins. ** *P* < 0.01, ****P* < 0.001.


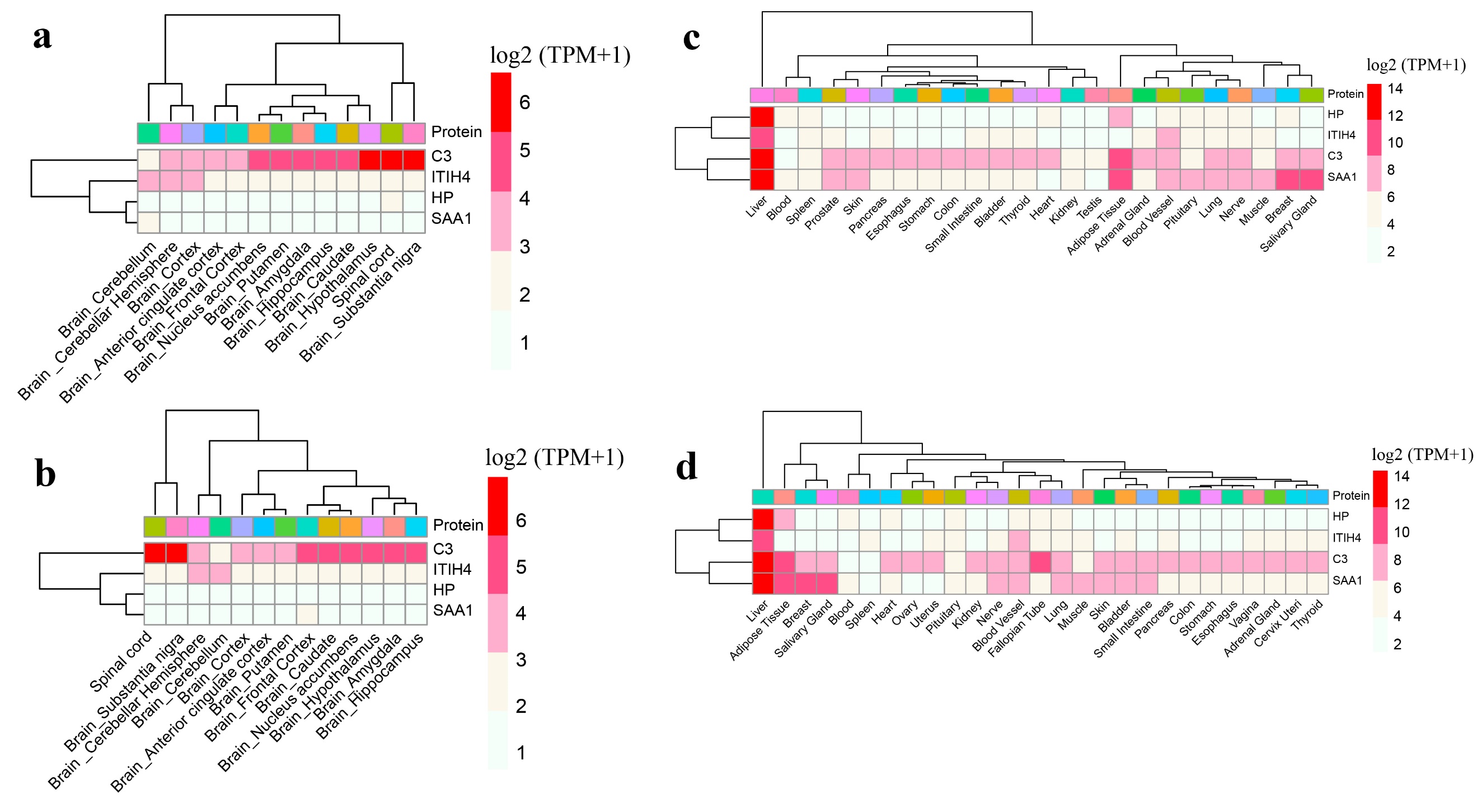


**Figure S2 Human multi-organ average expression levels for the coding genes of C3, HP, ITIH4, and SAA1 proteins**. Heatmap of average expression profiles for those coding genes in male (a) and female (b) brain regions. Heatmap of average expression profiles for those genes in male (c) and female (d) peripheral organs. Those Bulk-RNA seq data (GTEx_Analysis_2017-06-05_v8_RNASeQCv1.1.9_gene_tpm.gct) of brain regions and peripheral organs for human organ expression profiles, containing 11584 male and 5878 female samples, was obtained from GTEx database (<https://www.gtexportal.org/home/downloads/adult-gtex>). TPM, transcripts per million.


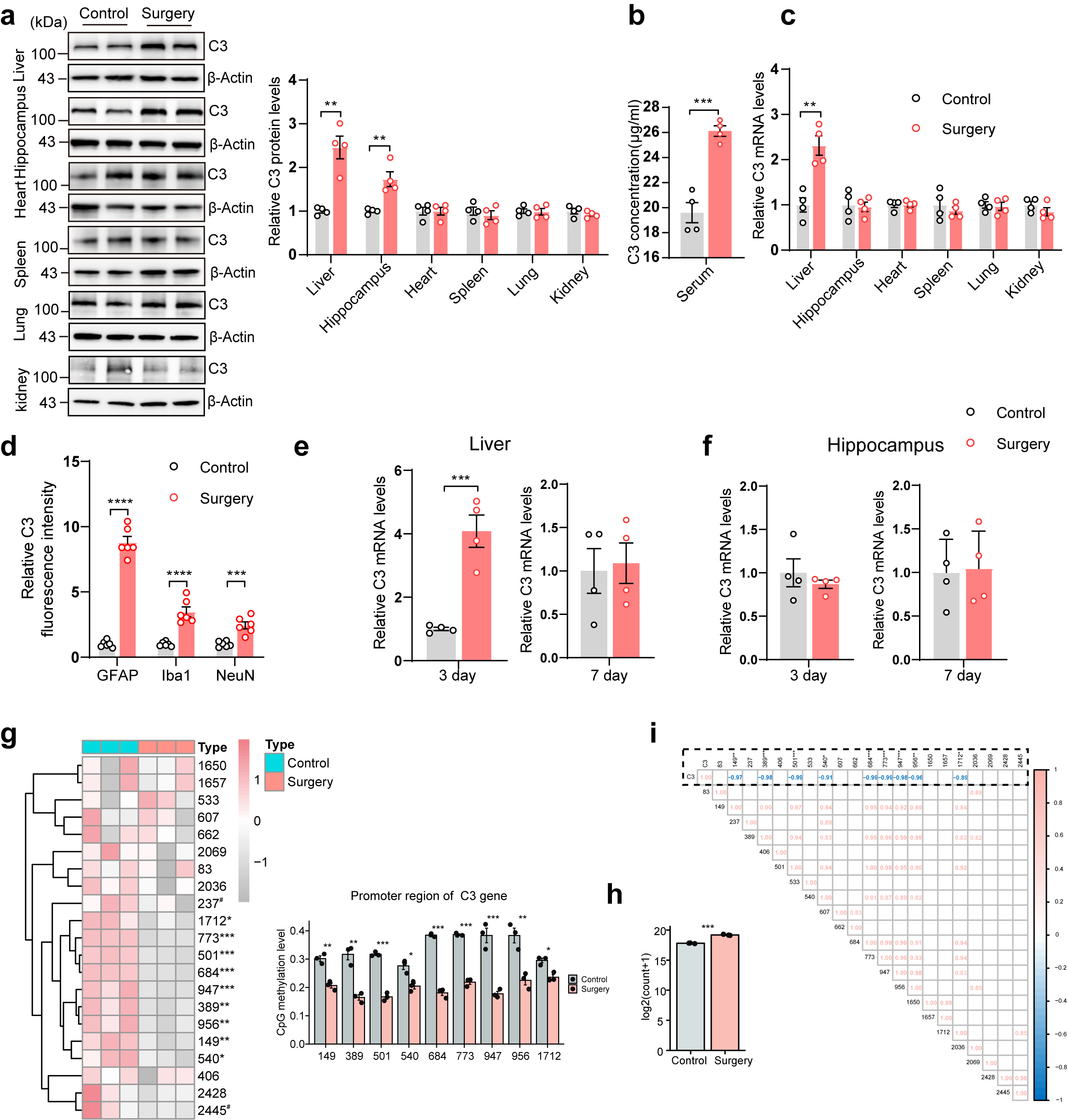


**Figure S3** **Anesthesia/surgery-induced molecular alterations and methylation changes of the *C3* promoter regions in multiple organs.** (a, b) Relative C3 mRNA and protein levels in the liver, hippocampus, kidney, heart, spleen and lung of 18-month-old mice 1d after tibial fractures internal fixation surgery (n=4 per group). (c) Serum C3 concentration changes in the aged mice 1d after tibial fractures internal fixation surgery (n=4). (d) The quantification for double-immunostaining of C3 (green) and GFAP (red), Neun(red), IBA1(red) in the hippocampal CA1 region from the control and surgery group (n =6 per group, the sample was obtained 1d after laparotomy). (e, f) Relative C3 mRNA levels in the liver and hippocampus of 18-month-old mice at 3d and 7d after laparotomy (n=4). (g) methylation levels of 9 CpG sites (149, 389, 501, 540, 684, 773, 947, 956, and 1712) in the C3 gene promoter in two groups of mice (n=3 per group). (h) Comparation of mRNA levels of C3 in the liver between control and surgery mice using Bulk-RNA sequencing (n=3 per group, the sample was obtained 1d after laparotomy). (i) Paired-sample Spearman correlation analysis between methylation levels of above 9 CpG sites and C3 mRNA levels. Statistical significance was determined using two-tailed unpaired Student’s t test. Data are presented as the means ± SEM. Asterisks indicate significant differences (**P* < 0.05, ***P* < 0.01, ****P* < 0.001).


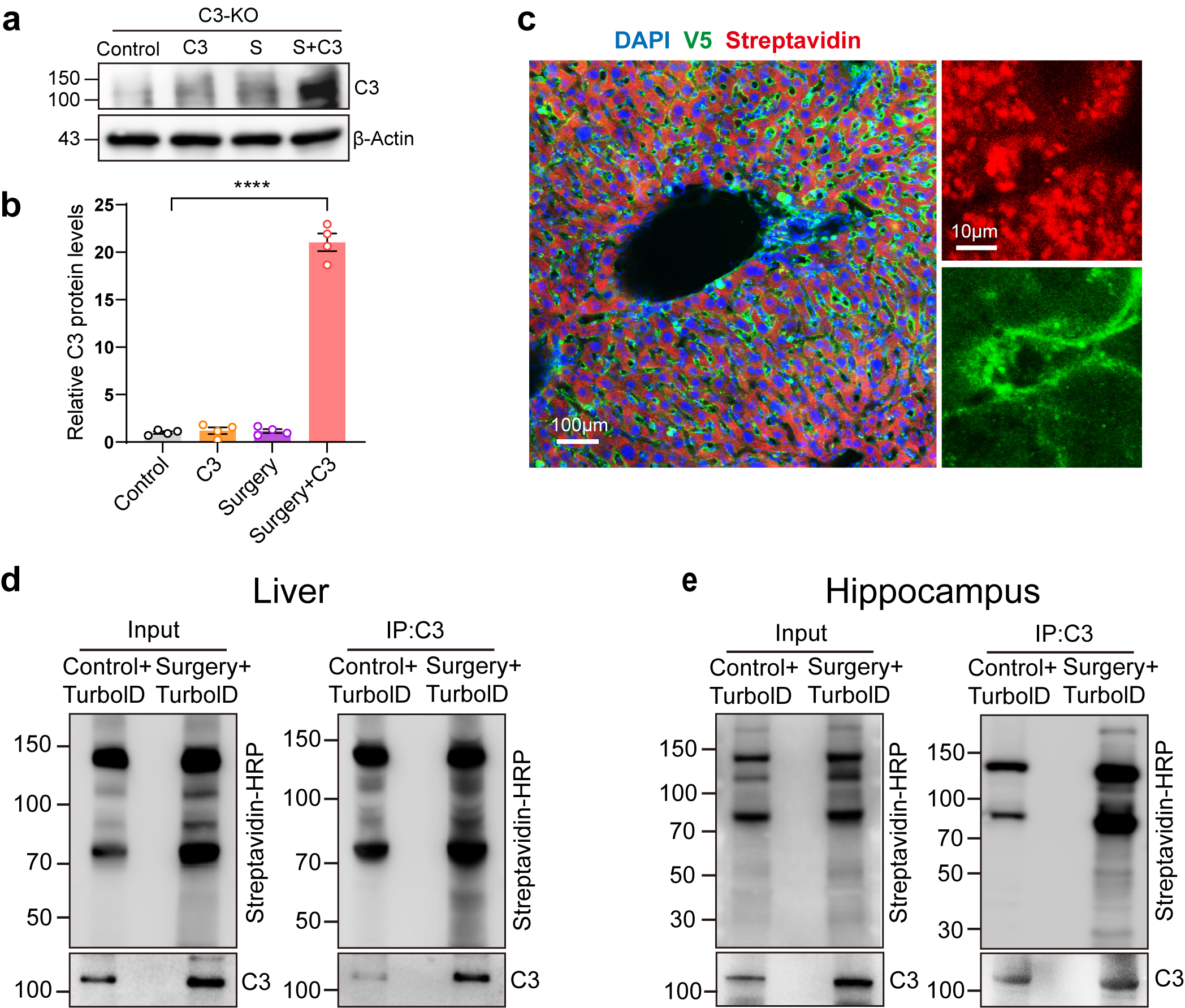


**Figure S4 Peripheral C3 could enter into the brain after anesthesia/surgery.** (a, b) Western blot of C3 protein levels in the hippocampus of C3-KO mice 6 h after tail vein injection of r-C3 protein. (c) Immunofluorescence imaging of TurboID (Anti-V5) and biotinylated proteins (Streptavidin-HRP) in the liver sections from Sec61b-V5-TurboID adenovirus (AdV) transduced mice. (d, e) Immunoprecipitation of hepatic biotinylated C3 in the liver (d) and hippocampus (e) from Sec61b-V5-TurboID adenovirus (AdV) transduced mice. Laparotomy was used as surgical method under anesthesia in mice. Statistical significance was determined using one-way ANOVA with the Dunnett's post-hoc test. Data are presented as the means ± SEM. *****P* < 0.0001. Scale bar was shown in the figure.


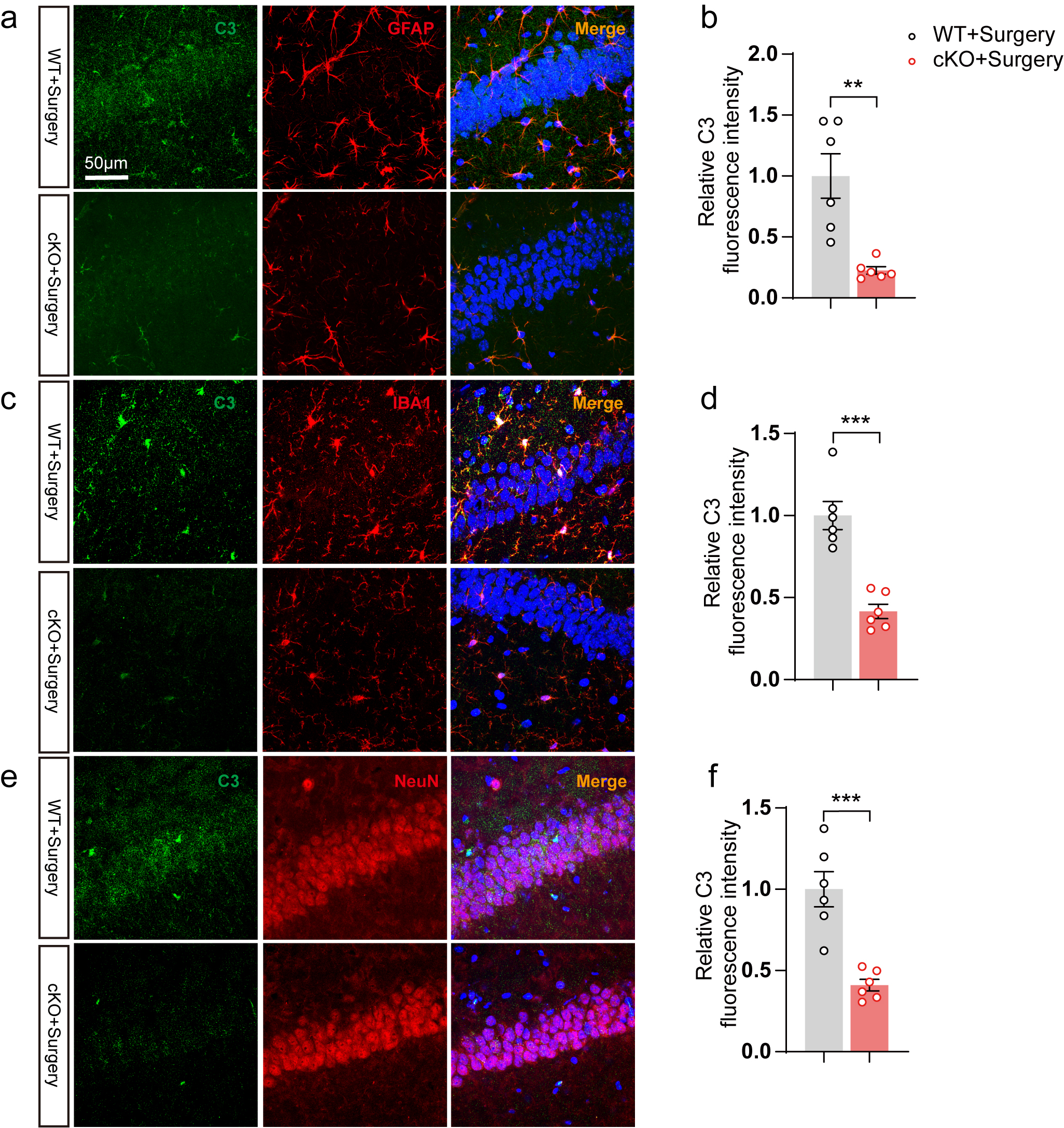


**Figure S5 The expression levels of C3 in hippocampal CA1 following anesthesia/surgery.** (a-f) Double-immunostaining of C3 (green) and GFAP (red), Neun (red), IBA1(red) in the hippocampal CA1 region from wild type (WT) and cKO mice after anesthesia/surgery (n =6 per group). Scale bars: 50 μm. Laparotomy was used as surgical method under anesthesia in mice. Wilcoxon test was used to determine the statistical significance of differences between two groups. Data are presented as the means ± SEM. ***P* < 0.01, ****P* < 0.001.


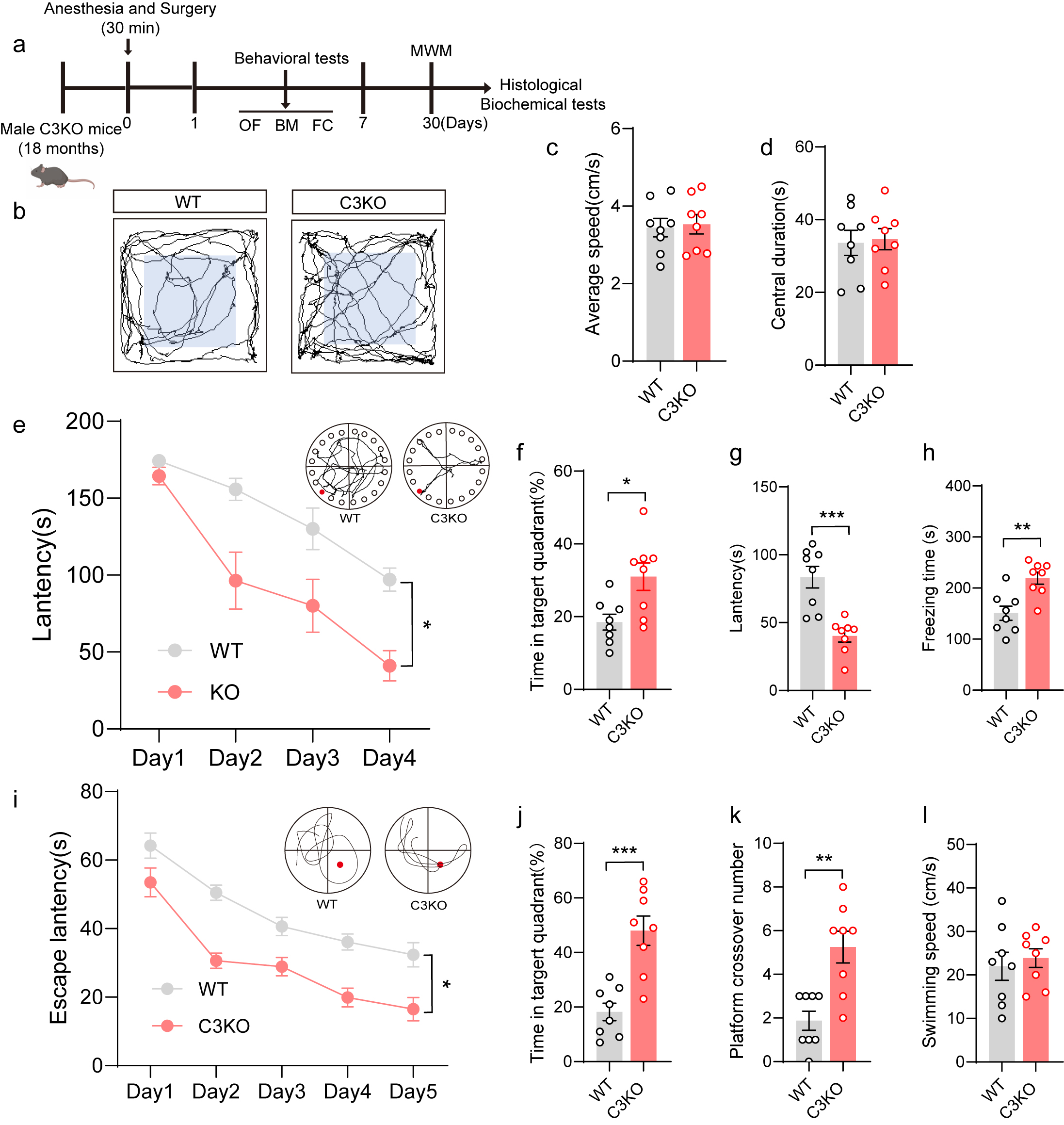


**Figure S6 The effect of C3 knockout on the cognitive function in aged mice.** (a) Schematic of the experimental design. (b) Representative tracking images of open field test between wild type (WT) and C3-KO mice. (c, d) Open field test including the average speed (c) and central duration (d) in WT and C3-KO mice (n = 8 per group). (e-g) Results of the Barnes maze tests of mice (n = 8 per group). Mean latency of mice in the training phase of 4 consecutive days (e). Time (%) spent in the target quadrant of mice in the testing phase (f). The latency of first appeared at the target (g). (h) Freezing time of mice in the fear conditioning test (n = 8 per group). (i-l) Results of the Morris water maze test (n = 8 per group). The latency to find the platform during the training stage between groups (i). The time spent in the target quadrant (j), crossover number (k) and swimming speed (l) in the target quadrant during the MWM testing stage (n = 8 per group). Laparotomy was used as surgical method under anesthesia in mice. Two-way repeated-measures ANOVA with Bonferroni's post hoc test for time × group comparisons. Student’s t test or Wilcoxon test was used to determine the statistical significance of differences between two groups. Data are presented as the means ± SEM. **P* < 0.05, ***P* < 0.01, ****P* < 0.001.


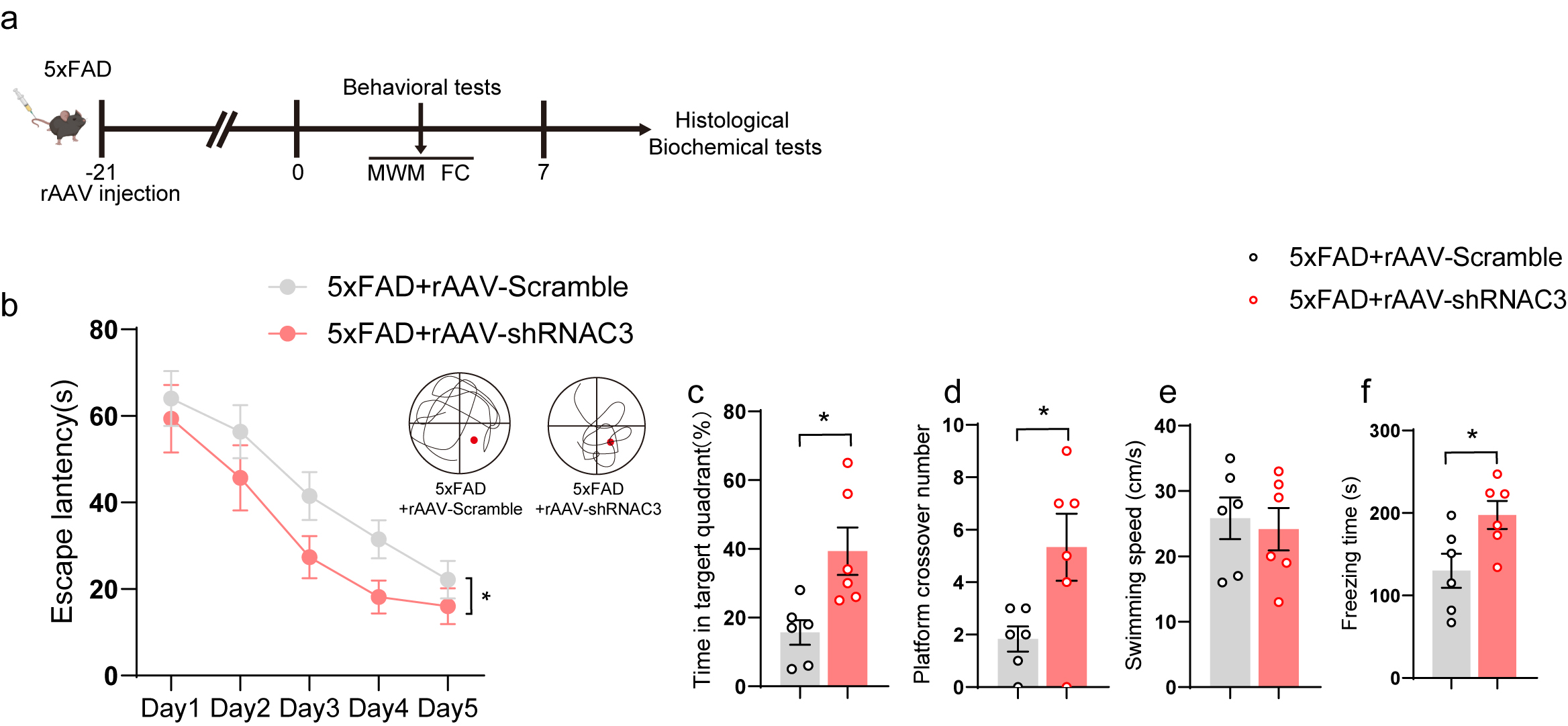


**Figure S7 Inhibition of liver-derived C3 alleviates cognitive impairment in 5×FAD mice.** (a) Schematic of the experimental design. (b-e) Results of the Morris water maze test in 5×FAD mice with or without knockdown of C3 (n = 6 per group). (b) The latency to find the platform during the training stage. (c-e) Results for the time spent in the target quadrant (c), crossover number (d) and swimming speed (e) in the target quadrant during the MWM testing stage. (f) Freezing time of 5×FAD mice with or without knockdown of C3 in the fear conditioning test (n = 6 per group). Two-way repeated-measures ANOVA with Bonferroni's post hoc test for time × group comparisons. Student’s t test or Wilcoxon test was used to determine the statistical significance of differences between two groups. Data are presented as the means ± SEM. **P* < 0.05.

**
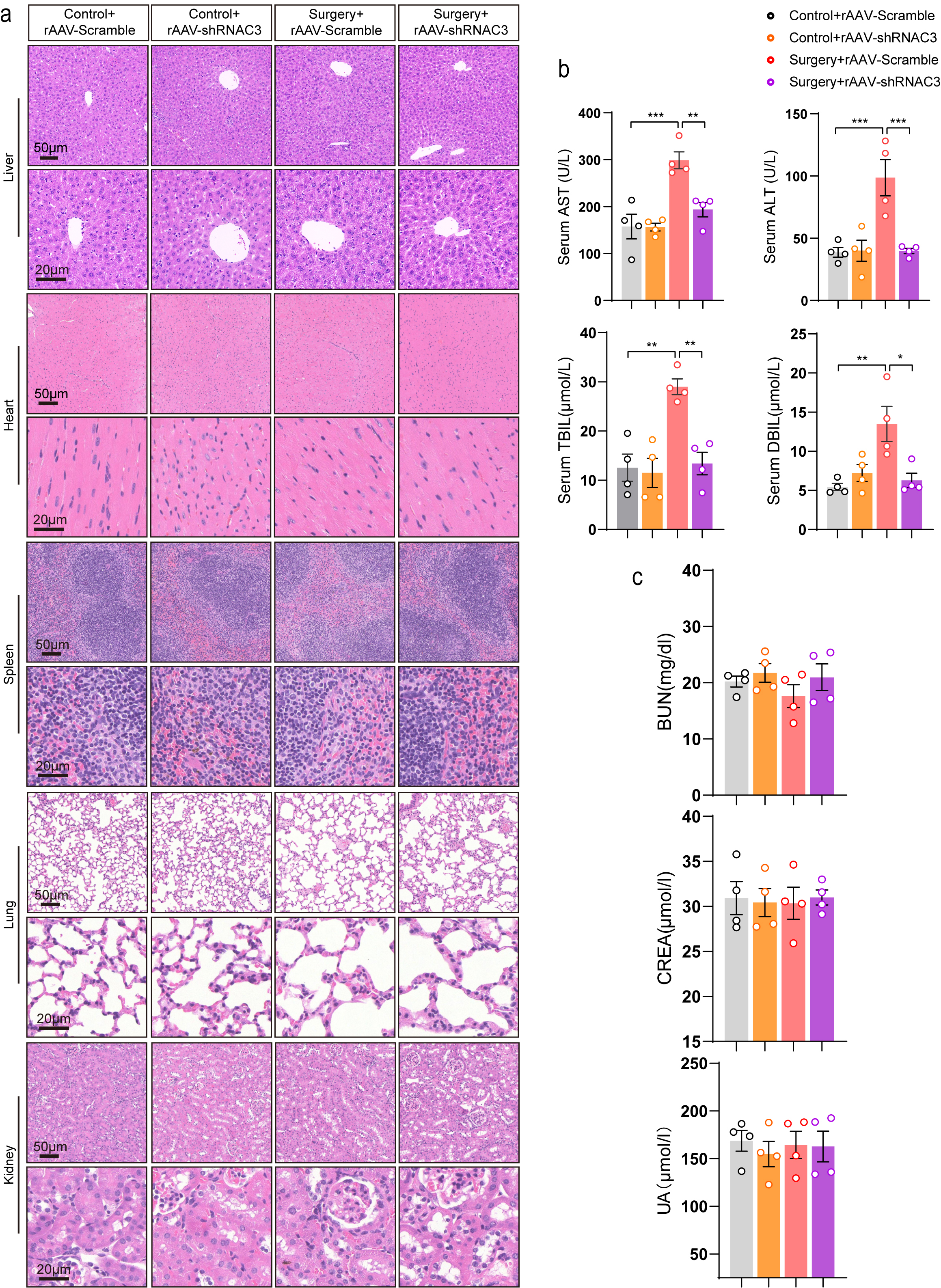
**

**Figure S8 Evaluation of the effects of anesthesia/surgery and rAAV-shRNAC3 virus on the body of mice.** (a) H&E of the heart, liver, spleen, lung and kidney sections from four groups of mice. (b) Comparison of serum alanine aminotransferase (ALT), aspartate aminotransferase (AST), total bilirubin (TBIL), and direct bilirubin (DBIL) among four groups. (c) Comparison of serum blood urea nitrogen (BUN), creatinine (CREA), and uric acid (UA) among four groups. Laparotomy was used as surgical method under anesthesia in mice. Statistical significance of four groups was determined utilizing one-way ANOVA with Tukey’s post-hoc test. Data are presented as the means ± SEM. **P* < 0.05, ***P* < 0.01, ****P* < 0.001. Scale bar was shown in the figure.


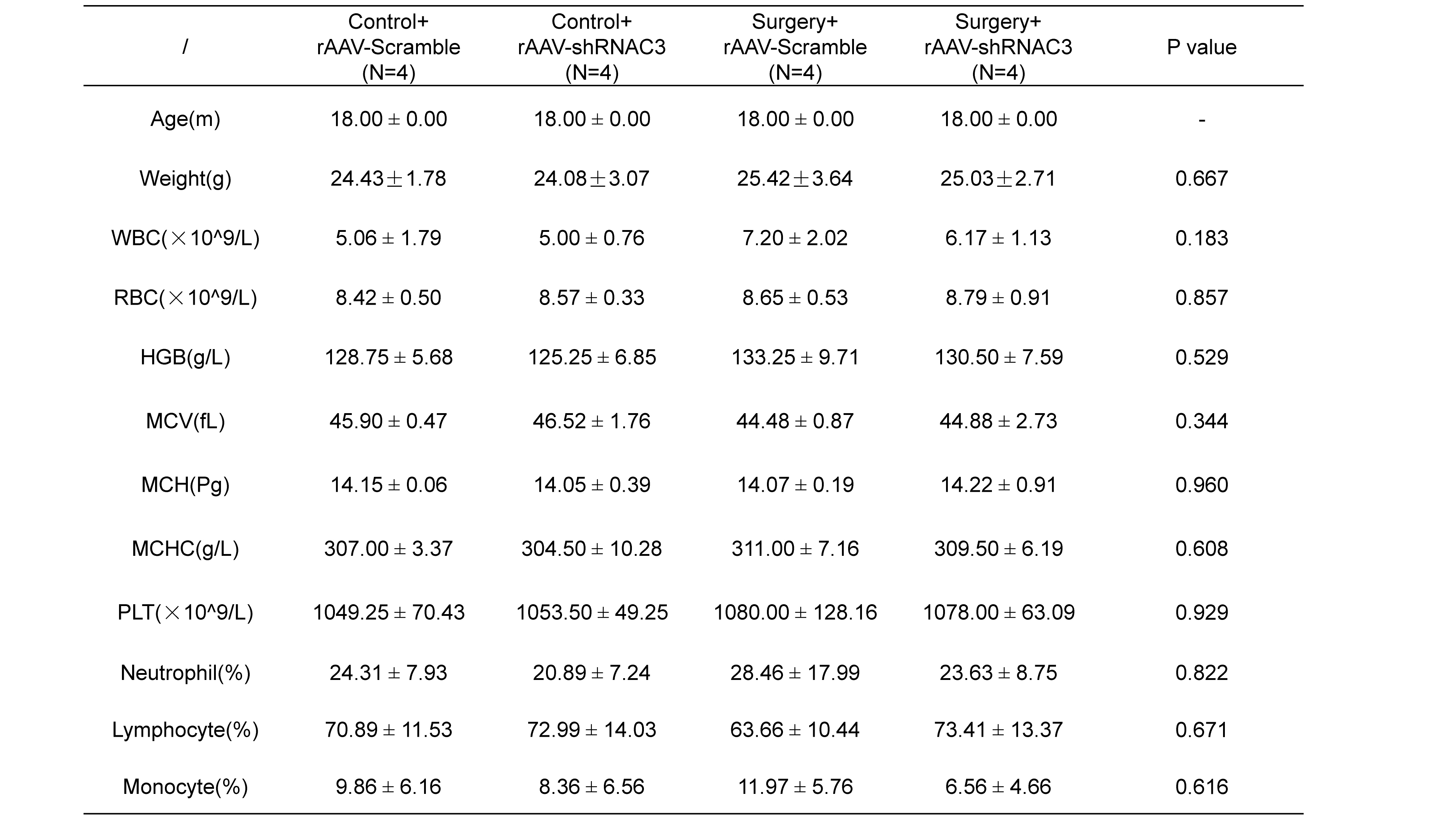


**Figure S9 Blood routine examination and physiological index between four groups of mice**. Laparotomy was used as surgical method under anesthesia in mice (n =4). Statistical significance was determined using one-way ANOVA with the Dunnett's post-hoc test. Data are presented as the means ± SEM. *P* value < 0.05 was considered statistically significant.


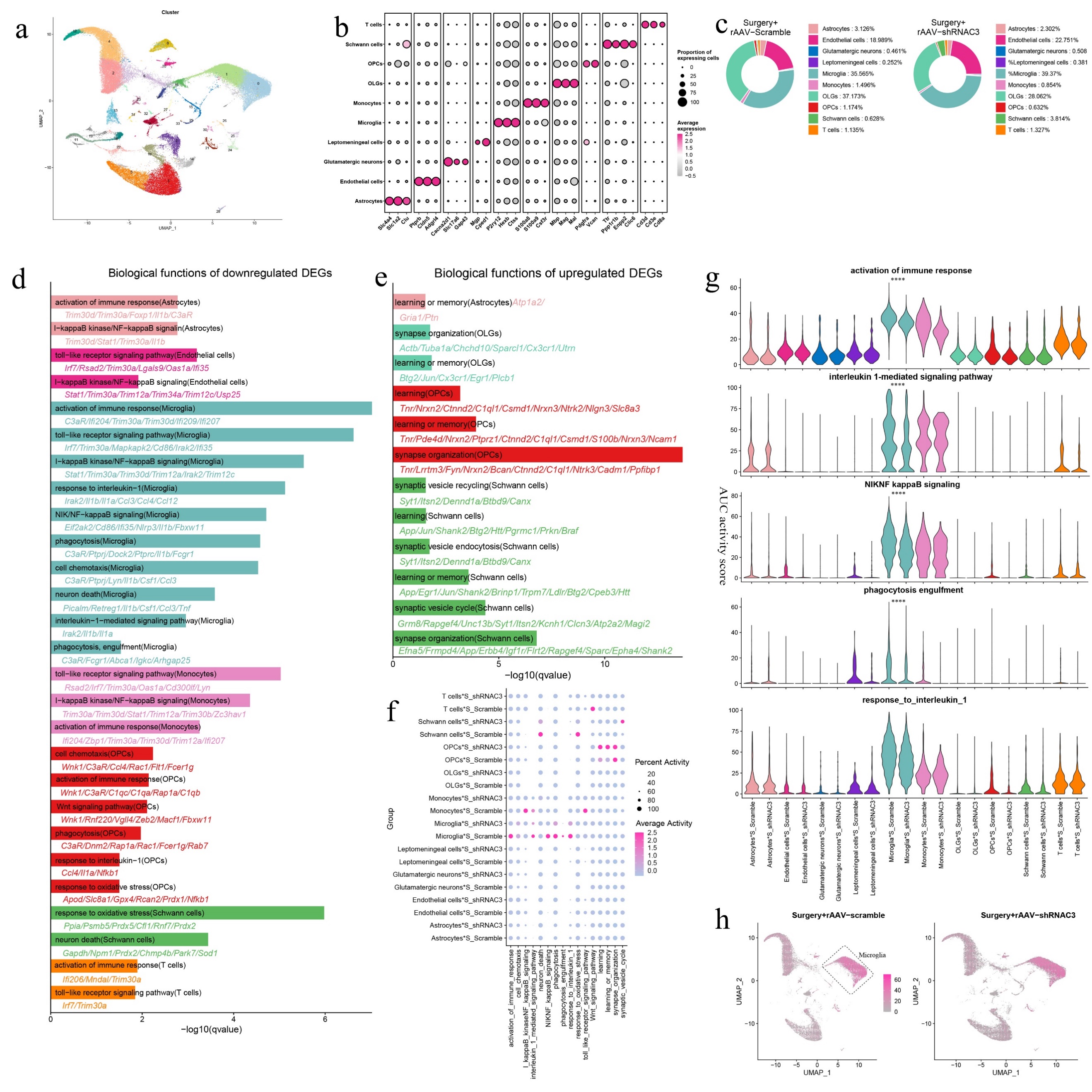


**Figure S10 Single-cell sequencing (scRNA-Seq) in the hippocampus between surgery mice injected with rAAV-Scramble and surgery mice injected with rAAV-shRNAC3**. (a) The UMAP visualization of 34 cell clusters including 83,565 hippocampal cells from two groups (n =3 per group). (b) Expression DotPlot of the known brain cell markers. (c) The proportion of 10 cell types between two groups. (d, e) Gene Ontology (GO) enrichment and KEGG pathway enrichment analyses for the downregulated (d) and upregulated (e) differentially expressed genes (DEGs). (f) DotPlot visualization of differential cognition-related terms based on the single-cell AUCell scores within each cell type. The |log (fold change)| > 0.25 and Benjamini-Hochberg adjusted *P* value < 0.05 were applied as the cutoff for screening differential terms. (g) Differential biological function analysis for phagocytic engulfment, NIK/NF-kappaB signaling, activation of immune response, interleukin 1-mediated signaling, and response to interleukin-1 based on the single-cell AUCell scores within each cell type. (h) The UMAP visualization of single-cell AUCell scores of phagocytosis in hippocampal cells between two groups. Laparotomy was used as surgical method under anesthesia in mice. *****P* < 0.0001.


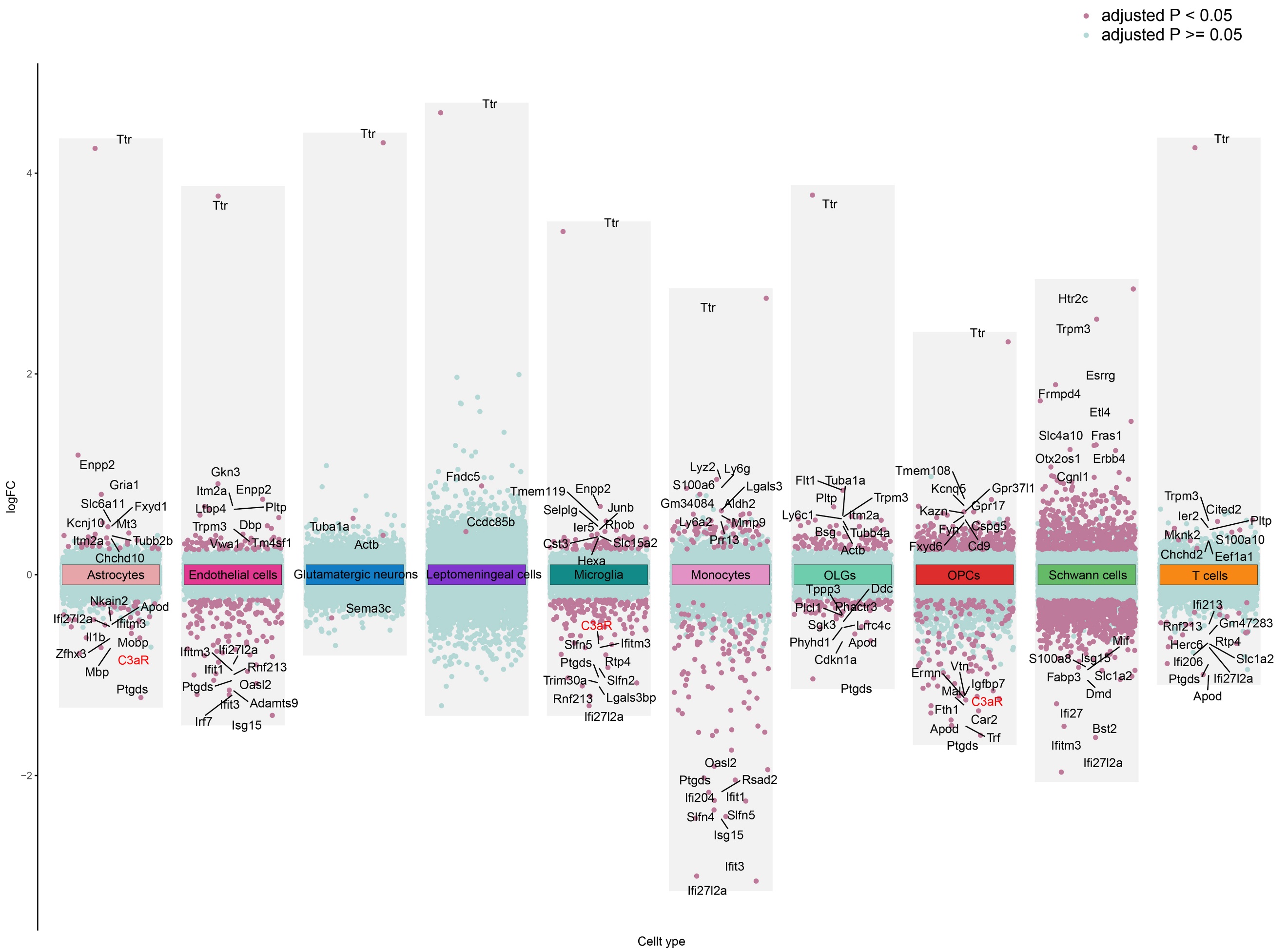


**Figure S11 Identification of differentially expressed genes (DEGs) in hippocampal cell types between surgery mice injected with rAAV-Scramble and surgery mice injected with rAAV-shRNAC3.** The |log2 (fold change)| > 0.25 and Benjamini-Hochberg adjusted *P* value < 0.05 was regarded as the cutoff.


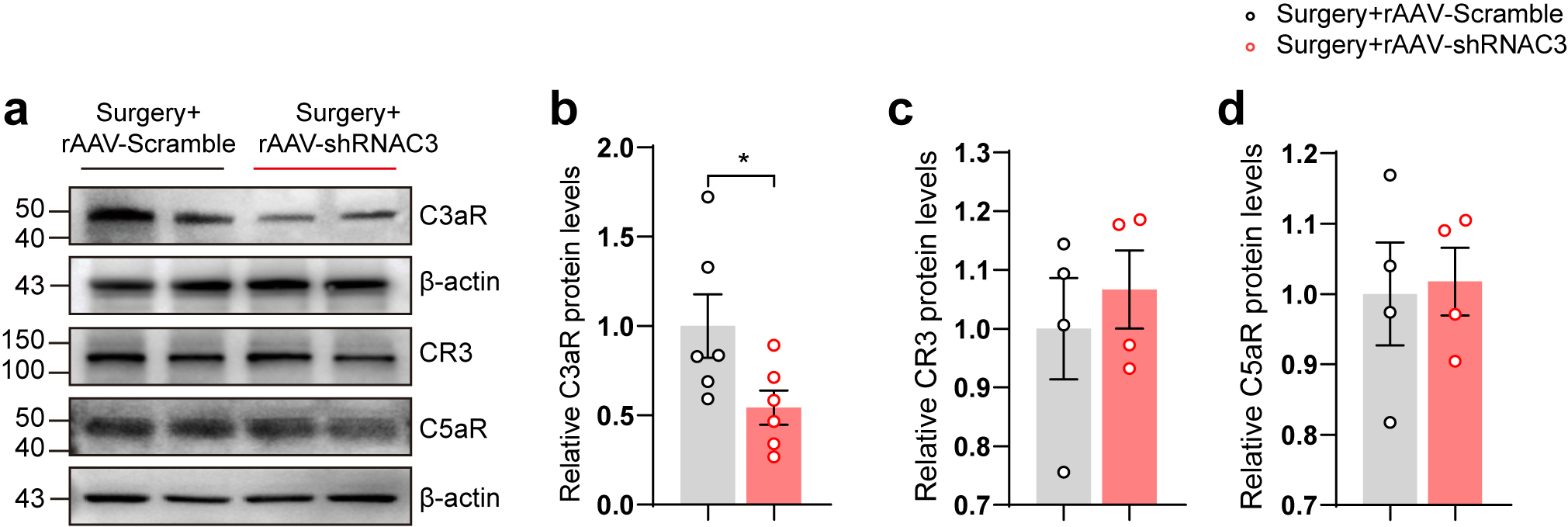


**Figure S12. Comparison of C3aR, CR3, and C5aR in the hippocampus between the mice injected with rAAV-Scramble and rAAV-shRNAC3 after anesthesia/surgery.** (a, b) Western blot of C3aR, CR3, and C5aR in the hippocampus of two groups (n = 6 per group). Laparotomy was used as surgical method under anesthesia in mice. Wilcoxon test was used to determine the statistical significance of differences between two groups. Data are presented as the means ± SEM. **P* < 0.05.


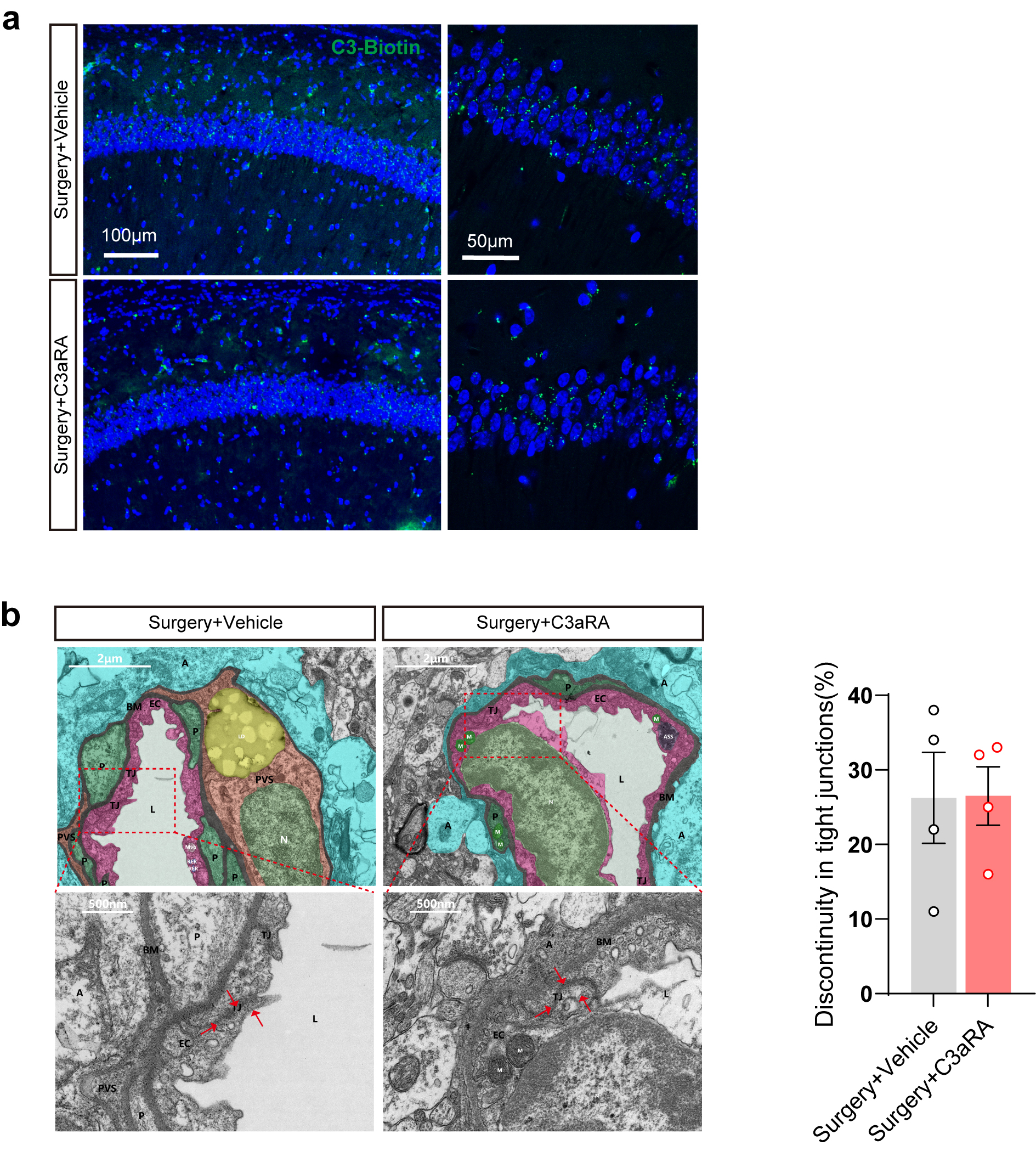


**Figure S13. The entry of peripheral C3 into the brain is required for anesthesia/surgery-activated C3-C3aR signaling.** (a) Immunofluorescent staining of peripheral biotinylated C3 in the hippocampus of control and surgery mice after vehicle and C3aRA (SB290157) treatment. (b) Transmission electron microscopy image of the BBB in the hippocampus of control or surgery mice after vehicle or C3aRA treatment. The red box highlights typical TJ gaps. Laparotomy was used as surgical method under anesthesia in mice. Wilcoxon test was used to determine the statistical significance of differences between two groups. Data are presented as the means ± SEM. *P* value < 0.05 was considered statistically significant. Scale bar was shown in the figure.

**Figure S14. Study design for the whole experiment.**


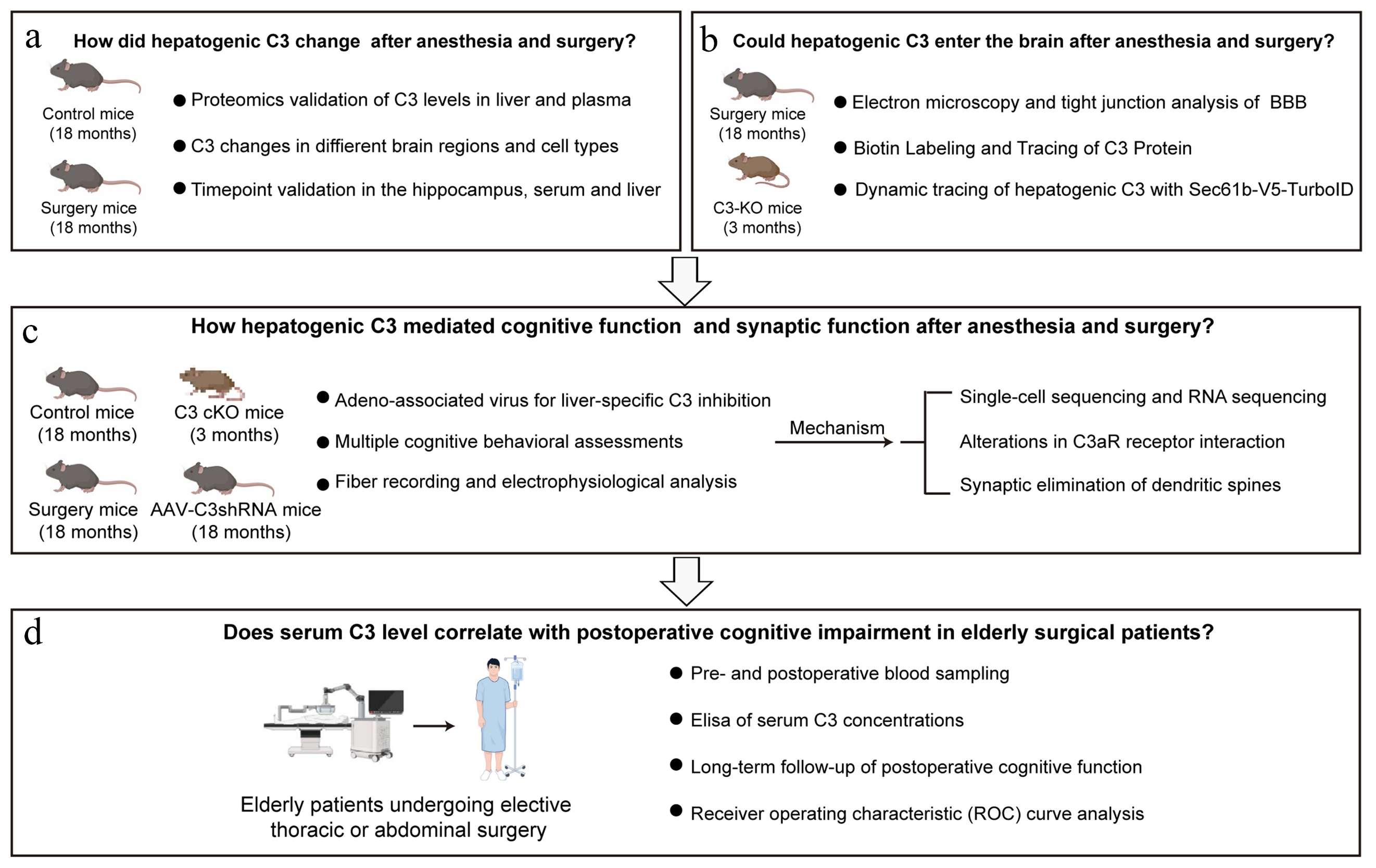


**Supplementary Table**

**Table S1** Hub proteins in the liver of determined by differential protein analysis and WGCNA for control and surgery mice.

**Table S2** Hub proteins in the plasma of determined by differential protein analysis and WGCNA for control and surgery mice.

**Table S3** 126 liver-derived blood proteins obtained from The Human Protein Atlas database.

**Table S4** Differentially methylation sites of C3 gene promotor in the liver between control and surgery mice.

**Table S5** Differentially expressed genes in the liver between control and surgery mice.

**Table S6** Hippocampal differentially expressed genes in the hippocampus between surgery mice injected with rAAV-Scramble and surgery mice injected with rAAV-shRNAC3 using scRNA-Seq.

**Table S7** Gene enrichment analysis of single-cell differentially expressed genes.

**Table S8** Single-cell differential analysis for AUCell scores of cognition-related biological function between surgery mice injected with rAAV-Scramble and surgery mice injected with rAAV-shRNAC3.

**Table S9** Differentially expressed genes in the hippocampus between surgery mice injected with rAAV-Scramble and surgery mice injected with rAAV-shRNAC3 obtained by Bulk-RNA Seq.

**Table S10** Clinical information of surgical patients.

**Table S11** The primers of C3 gene promotor.
